# The chloride intracellular channel 1 (CLIC1) is essential for microglial morphodynamics and neuroinflammation

**DOI:** 10.1126/sciadv.ads9181

**Published:** 2025-10-22

**Authors:** Ali Rifat, Tom Bickel, Patricia Kreis, Thorsten Trimbuch, Julia Onken, Andranik Ivanov, Giulia Albertini, Dieter Beule, Michele Mazzanti, Harpreet Singh, Britta J. Eickholt, Bart De Strooper, Jörg R. P. Geiger, Christian Madry

**Affiliations:** ^1^ Institute of Neurophysiology, Charité - Universitätsmedizin Berlin, corporate member of Freie Universität Berlin and Humboldt-Universität zu Berlin, Charitéplatz 1, 10117 Berlin, Germany.; ^2^ Berlin Institute of Health at Charité - Universitätsmedizin Berlin, Charitéplatz 1, 10117 Berlin, Germany.; ^3^ Institute of Biochemistry and Molecular Biology, Charité - Universitätsmedizin Berlin, corporate member of Freie Universität Berlin and Humboldt-Universität zu Berlin, Charitéplatz 1, 10117 Berlin, Germany.; ^4^ Department of Neurosurgery, Charité - Universitätsmedizin Berlin, corporate member of Freie Universität Berlin and Humboldt-Universität zu Berlin, Charitéplatz 1, 10117, Berlin, Germany.; ^5^ Core Unit Bioinformatics, Berlin Institute of Health at Charité - Universitätsmedizin Berlin, Berlin, Germany.; ^6^ Centre for Brain and Disease Research, Flanders Institute for Biotechnology (VIB), Leuven, Belgium.; ^7^ Department of Neurosciences and Leuven Brain Institute, KU Leuven, Leuven, Belgium.; ^8^ Laboratory of Cellular and Molecular Physiology, Department of Biosciences, University of Milan, Via Celoria 26, I-20133 Milano, Italy.; ^9^ Department of Physiology and Cell Biology, College of Medicine, The Ohio State University Wexner Medical Center, Columbus, OH, USA.; ^10^ UK Dementia Research Institute at UCL, University College London, London, UK.

## Abstract

Microglial functions rely on their morphodynamic versatility and inflammatory response, yet the molecular determinants, particularly ion channels and receptors, remain poorly understood. Here, we identify chloride intracellular channel 1 (CLIC1), a protein known to exist in both soluble and membrane-associated forms, as highly enriched in human and murine microglia, with minimal expression in other brain cells. Acute blockade or genetic deletion of CLIC1 markedly attenuates microglial surveillance by reducing ramification and motility, without affecting chemotaxis. This phenotype is recapitulated in xenografted human microglia and human brain tissue. Mechanistically, CLIC1 effects involve interactions with actin-binding ezrin, radixin, and moesin (ERM) proteins, suggesting a role in linking the plasma membrane to the cytoskeleton. Contrary to its name, CLIC1 functions are chloride-independent and thus unlikely to reflect ion channel activity. This is supported by patch-clamp electrophysiology revealing lack of chloride conductance in surveillant microglia. Following ATP–evoked activation, CLIC1 blockade strongly suppresses NLRP3–dependent interleukin-1β release, suggesting therapeutic potential against neuroinflammation.

## Teaser

CLIC1 regulates surveillance, morphology, and interleukin release in mouse and human brain microglia.

## INTRODUCTION

The ability of microglia to react effectively to physiological and pathophysiological challenges is essential for their protective role in the brain and ability to generate an adequate immunological response. One of the key requirements for ensuring their multiple functions relates to the enormous dynamics of microglia, enabling constant surveillance of the brain parenchyma to monitor neural function and detect pathological insults, thereby maintaining brain homeostasis (*1*, *2*).

Microglial surveillance depends on morphological criteria, mainly the ramification and length of their processes, and on dynamic properties, i.e., the speed and frequency of process extensions and retractions and, for cerebellar microglia, also somatic displacements (*3*, *4*). Apart from nondirected surveillance, microglial processes can also move in a highly targeted manner toward chemotactic stimuli such as increases in ambient adenosine 5ʹ triphosphate (ATP) levels (*5*). These properties, collectively referred to as morphodynamics, are markedly shaped by ion channels and membrane receptors, the expression of which varies depending on the state of microglial activation (*6*). While chemotaxis is controlled by purinergic P2Y12 receptor signaling, the ramification and surveillance of microglia depend on the resting membrane potential set by the tonic activity of tandem pore domain halothane-inhibited potassium channel 1 (THIK-1) K^+^ channels (*2*, *7*). Ultimately, microglial motility is driven by continuous remodeling of the cytoskeleton and polymerization of actin filaments (*8*–*11*), but the mechanisms by which ion channels and receptors regulate these processes are largely unknown. In other cell types, several ion channels, many of them anion-permeable, have been shown to interact with actin (*12*), raising the possibility of similar regulatory functions in microglia.

A deeper understanding of the function and associated molecular identity of many microglial ion channels is still lacking, despite major advances in their genetic profiling and the mapping of ion channel–encoding genes to specific microglial subtypes and activation states. This applies particularly to the Cl^−^ ion channel family, a structurally and functionally diverse class of anion-selective channels, of which volume-regulated Cl^−^ channels (VRACs), Ca^2+^-activated Cl^−^ channels (CaCCs), and chloride intracellular channels (CLICs) have been described in microglia (*13*). Many of these findings were obtained from cell lines or primary cultures, the genetic profile and functional state of which differ substantially from those of native microglia in brain tissue (*14*), for which only a few studies exist (*8*, *15*–*18*). Cl^−^ channels have been implicated mainly in activated microglia or upon disturbance of brain homeostasis, and nonspecific Cl^−^ channel blockers [such as 4,4′-diisothiocyanato-2,2′-stilbenedisulfonic acid disodium salt (DIDS), 5-nitro-2-(3-phenylpropylamino)benzoic acid (NPPB), or fenamates] or impairment of transmembrane Cl^−^ gradient have been shown to affect microglial ramification and process movement (*8*, *18*–*20*). Chloride conductance may also contribute to regulating surveillance by counterbalancing hyperpolarizing tonic THIK-1 activity. This is due to the rather positive resting potential of ~−40 mV of nonactivated microglia, which suggests the presence of an additional depolarizing conductance (*6*, *21*). Chloride could play a role, given that the Cl^−^ equilibrium potential of cortical microglia is in the range of their resting potential, reflecting their rather high intracellular Cl^−^ concentration (*20*). Collectively, these findings suggest a role for Cl^−^ channels in the regulation of microglial morphodynamics and other Cl^−^-dependent functions, but the molecular determinants of such effects and their implications under physiological conditions remain to be elucidated.

Here, we identify chloride intracellular channel 1 (CLIC1) as the most highly expressed member of the Cl^−^ channel–related family in microglia in healthy mouse and human brains. Using live multiphoton imaging and morphological characterization combined with pharmacological and genetic interventions, we show that CLIC1 is essential for maintaining the surveillance and ramification of microglia. We further show that these functions are independent of the extracellular Cl^−^ concentration and mechanistically converge with effects induced by proteins of the ezrin, radixin, and moesin (ERM) family, acting as molecular linkers between the plasma membrane and the actin cytoskeleton (*22*). Consistent with an ion channel–independent role of CLIC1, patch-clamp electrophysiology reveals a lack of plasma membrane Cl^−^ conductance in unchallenged microglia, suggesting that Cl^−^ channel activity per se is dispensable for maintaining surveillance. We also show via immunological assays that CLIC1 is critical for the NOD-like receptor pyrin domain containing 3 (NLRP3)–driven release of interleukin-1β (IL-1β) upon purinergic activation of mouse and human brain microglia.

## RESULTS

### Broad-spectrum Cl^−^ channel blockers suppress microglial surveillance in the absence of plasma membrane Cl^−^ channel activity

**Fig. 1.**
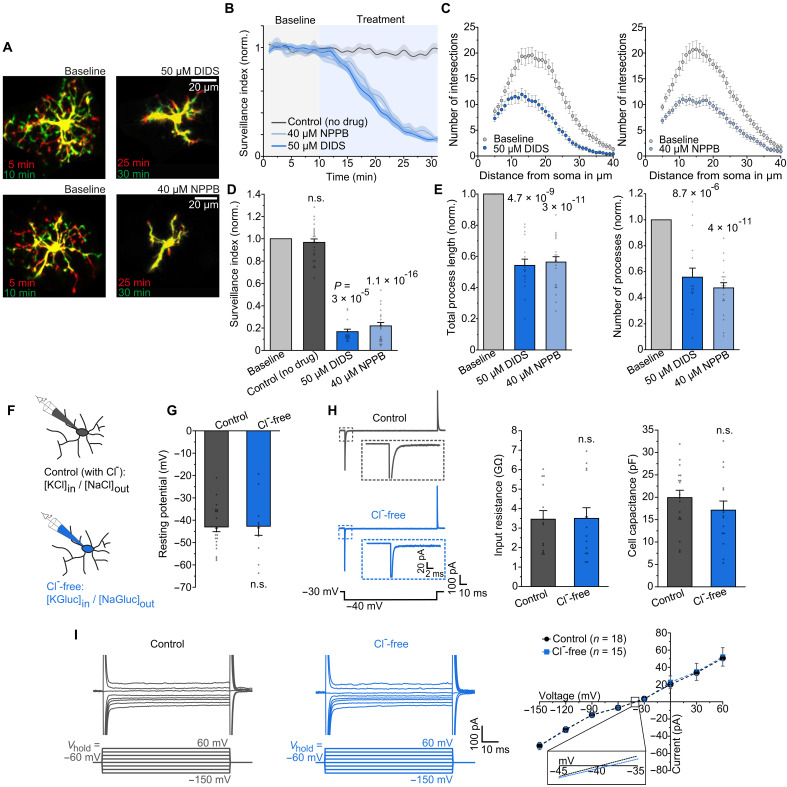
Broad-spectrum Cl^−^ channel blockers suppress microglial surveillance in the absence of Cl^−^ channel activity. (**A**) Superimposed images of microglia captured 5 min apart before (baseline) and with DIDS and NPPB, illustrating process extensions (green), retractions (red), and immobile regions of the cell (yellow) during surveillance. (**B**) Surveillance of microglia for control and drug treatment, normalized to the first 10 min of the baseline. (**C**) Sholl analysis–derived number of intersections, comparing the ramification of cells prior to and during application of DIDS (left) and NPPB (right). (**D**) Mean effects of Cl^−^ channel blockers DIDS (*n* = 16) and NPPB (*n* = 21) compared with the control (no drug; *n* = 26) on surveillance (average of the last 5 min per condition), normalized to the baseline (average of the first 10 min). (**E**) Quantification of Sholl analysis–derived total process length (left; *n* = 17 and 22) and number of processes (right; *n* = 16 and 22), normalized to the baseline (before drug application). (**F**) Schematic of patch-clamped microglia in the presence (gray) and absence (blue) of Cl^−^, the latter abolishing any influence of Cl^−^ on the electrical membrane properties (see Materials and Methods). (**G**) Quantification of the resting membrane potential (determined immediately after gaining electrical access to the cell) for control (*n* = 21) and Cl^−^ free (*n* = 11) conditions. (**H**) Membrane currents in response to 10-mV hyperpolarizing voltage steps (left) from which input resistance (middle; *n* = 14 each) and cell capacitance (right; *n* = 18 and 15) were determined depending on Cl^−^ abundance. (**I**) Specimen current profiles in response to voltage steps ranging from −150 mV to +60 mV (left) and corresponding mean voltage dependence of currents per condition (right). Data information: Data indicate means ± SEM. Dots on bars show number of cells. Data are from three mice per experimental condition. *P* values are from paired [(D) and (E)] and unpaired [(G) and (H)] Student’s *t* tests. n.s., not significant.

To test the involvement of Cl^−^ channels in the regulation of microglial surveillance, we applied two widely used broad-spectrum Cl^−^ channel inhibitors, DIDS and NPPB (*23*), to acute cortical brain slices from adult *Cx3cr1*^eGFP/+^ reporter mice (*24*) and live imaged microglia via multiphoton microscopy (Fig. 1A). While surveillance remained constant under baseline conditions, with frequent process extensions and retractions, exposure to DIDS and NPPB induced a strong reduction in surveillance and ramification (Fig. 1, B to E, and movies S1 and S2). As both substances act on a wide range of Cl^−^ channels (*23*), this suggests the presence of Cl^−^ conductance affecting the electrical membrane properties of microglia, with the resting potential being an important determinant of microglial surveillance (*7*). To study chloride channel activity directly, we used electrophysiology and patch-clamped cortical microglia in the presence of Cl^−^ and under Cl^−^-free conditions, using isotonic intra- and extracellular [artificial cerebrospinal fluid (aCSF)] solutions in which Cl^−^ was replaced by gluconate (Fig. 1F). Unexpectedly, the resting membrane potential of microglia was not affected in slices 30-min preequilibrated in Cl^−^-free aCSF and remained unchanged over time (Fig. 1G and fig. S1A), indicating a lack of Cl^−^ channel activity in the plasma membrane of microglia. Consistently, no differences were observed in input resistance, cell capacitance, and time- and voltage-dependent membrane currents in the absence of Cl^−^ (Fig. 1, H and I). However, microglia generated outwardly rectifying membrane currents to hypotonic extracellular solution that required the presence of Cl^−^, suggesting activation of swelling-induced chloride channels upon disturbance of brain homeostasis by osmotic stress (fig. S1, A and B). These results show that the potent inhibition of microglial surveillance by nonselective Cl^−^ channel blockers is not due to Cl^−^ channel activity in the plasma membrane, suggesting the involvement of intracellular Cl^−^ channels or other, nonelectrogenic mechanisms.

### *Clic1* is the most highly expressed chloride channel in mouse and human microglia

**Fig. 2.**
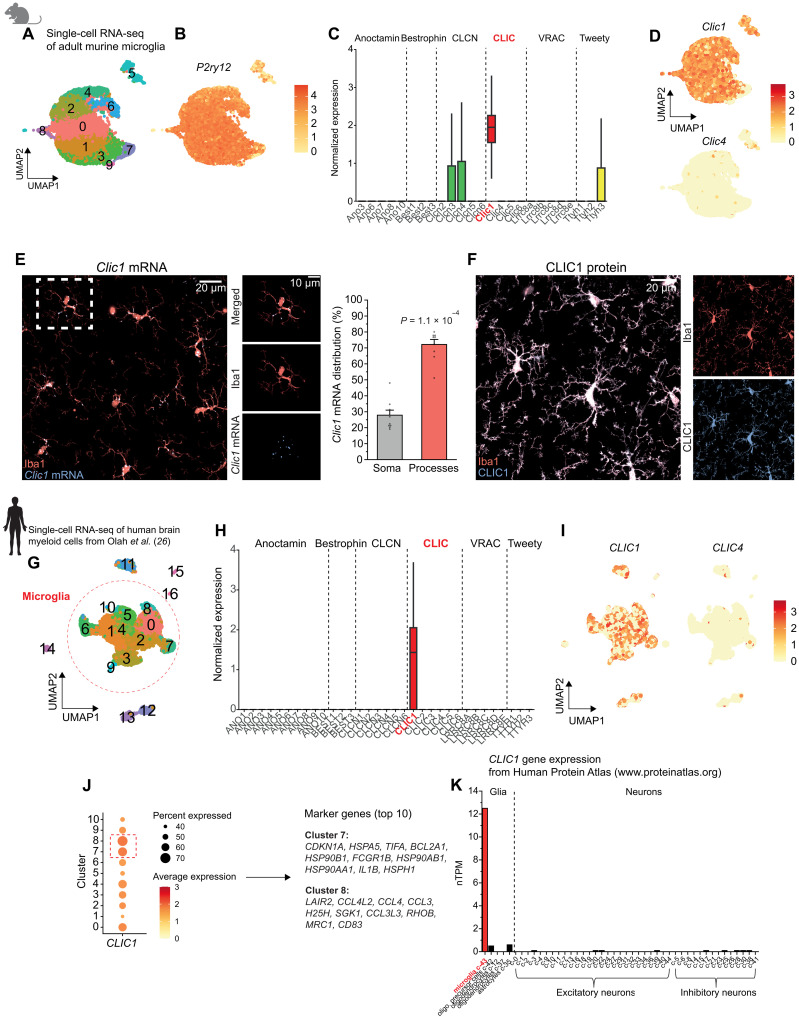
*Clic1* is the highest expressed Cl^−^ channel in murine and human microglia. (**A** and **B**) Uniform manifold approximation and projection (UMAP) from single-cell RNA sequencing (RNA seq) of 9907 mouse microglia displaying (A) identified clusters and (B) *P2ry12* gene expression in all clusters indicating microglia specificity. (**C**) Pseudo-bulk expression profiles of genes of different Cl^−^ channel families. (**D**) UMAPs of *Clic1* and *Clic4* gene expression across individual microglia, revealing high and ubiquitous expression of *Clic1*. (**E**) Left: Specimen overview and close-up images visualizing *Clic1* mRNA by RNAscope in situ hybridization colabeled for microglia by Iba1 immunofluorescence in mouse brain slices. *Clic1* mRNA signal is shown as colocalization with microglia defined by Iba1 staining. Right: Quantification of *Clic1* mRNA puncta reveals enrichment in microglial processes over somata (*n* = 9). (**F**) Specimen images showing abundant distribution of CLIC1 immunofluorescence (protein) within Iba1-positive microglial somata and processes. Only CLIC1 signal colocalizing with Iba1 is shown, as CLIC1 is also expressed in endothelial cells (*28*, *38*). (**G**) UMAP from single-cell RNA sequencing of human brain myeloid cells using data from Olah *et al.* (*26*), depicting 17 identified clusters, 11 of which are microglia (red circled). (**H**) As for (C) but in human microglia. (**I**) UMAPs illustrating *CLIC* gene expression in human microglia. (**J**) Left: *CLIC1* expression among microglia clusters, indicating enrichment in clusters 7 and 8. Right: Top 10 marker genes for clusters 7 and 8, including genes involved in inflammation (e.g., *IL1B*) and actin cytoskeleton organization (e.g., *RHOB*); see also table S1. (**K**) Analysis of *CLIC1* gene expression using data from the Human Protein Atlas (www.proteinatlas.org) in different brain cell types, comprising glial cells and excitatory and inhibitory neurons. Data in (A) to (E) are from three mice. See Olah *et al.* (*26*) for detailed information on the human transcriptome data [(G) to (J)]. *P* value is from one-sample Student’s *t* test (E). TPM, transcripts per million.

To elucidate the molecular determinants underlying the DIDS- and NPPB-induced effects, we investigated the expression profiles of genes encoding the different chloride channel families in microglia from adult mice. Single-cell RNA sequencing resulted in 10 distinct microglial clusters positive for the cell type–specific marker gene *P2ry12* (Fig. 2, A and B). Pseudo-bulk and single-cell analyses of the transcriptomic data revealed the expression of voltage-gated chloride channels, CLICs, VRACs, and CaCCs of the anoctamin and tweety families (Fig. 2, C and D, and fig. S2A). Among all the identified transcripts, *Clic1* was by far the most highly and ubiquitously expressed gene in murine microglia.

To obtain insights into the subcellular distribution of *Clic1* transcript, we visualized individual mRNA molecules by RNAscope in situ hybridization in mouse brain slices colabeled for microglia by Iba1 immunofluorescence. This revealed significant accumulation of *Clic1* mRNA in microglial processes as compared to the cell somata, yielding a ratio of almost 3:1 (Fig. 2E). In line with this, CLIC1 protein was abundantly expressed in processes and somata of microglia, as demonstrated by almost complete colocalization of Iba1 and CLIC1 immunoreactivity (Fig. 2F and fig. S2, B and C). Supporting these findings, data from a recent study by Vasek *et al.* (*25*), which used subcellular ribosome affinity purification, revealed *Clic1* to be a highly enriched and locally translated transcript within peripheral microglial processes (fig. S2D).

Similarly, analysis of sequencing data from the human brain (*26*) revealed high and nearly exclusive expression of *CLIC1* mRNA in microglia at the pseudo-bulk and single-cell levels (Fig. 2, G to K). Apart from sparsely expressed *CLIC4* in very few cells, no other *CLIC* genes were found in human or murine microglia (Fig. 2, C, D, H, and I). While *CLIC1* was abundant in most human clusters, particularly high levels of *CLIC1* were detected in clusters 7 and 8, which comprise marker genes involved in the regulation of cell motility, actin cytoskeleton organization, and inflammatory responses (Fig. 2J and table S1). Compared with other glial cells and neurons, *CLIC1* is expressed almost exclusively in microglia in the human brain (Fig. 2K) (*27*). These data, which identify *Clic1* as the most highly expressed chloride channel in human and mouse microglia under physiological conditions, suggest a role for CLIC1 in global microglial functions.

### CLIC1 regulates microglial surveillance by affecting ramification and motility of cellular processes

**Fig. 3.**
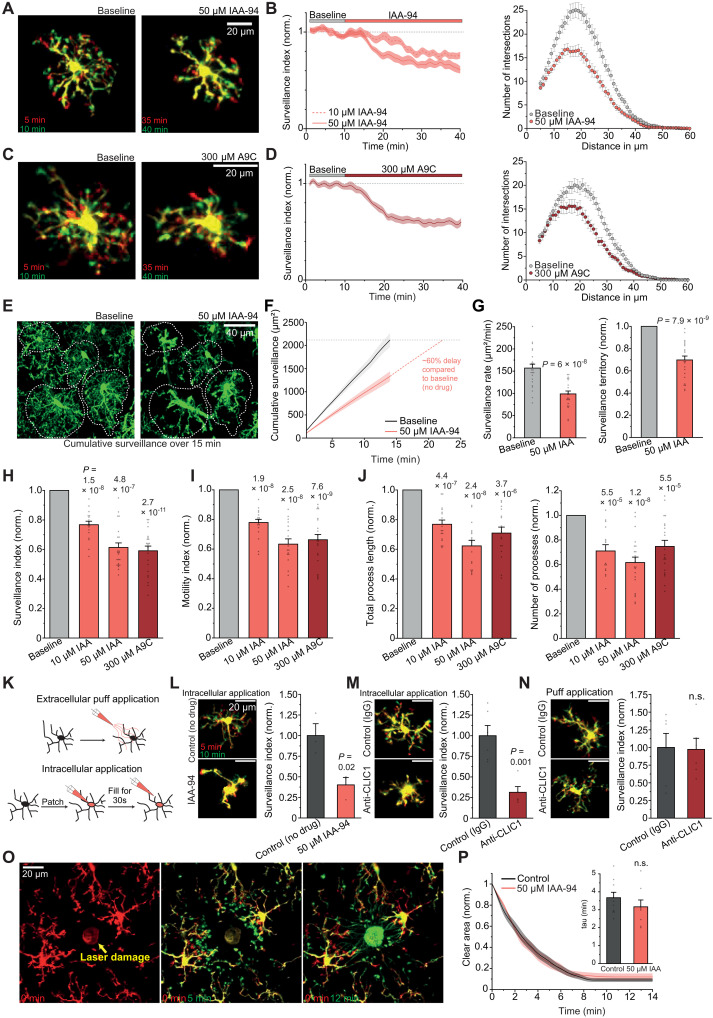
Effect of CLIC1 blockade on microglial morphodynamics. (**A**) Superimposed images of microglia, before and with IAA-94. (**B**) Left: Time course of microglial surveillance before and with 10 and 50 μM IAA-94, normalized to baseline. Right: Ramification by Sholl analysis before and with IAA-94. (**C** and **D**) As for (A) and (B) but with application of A9C. (**E**) Superimposed images of cumulatively surveilled area over 15 min before and with IAA-94. (**F**) Cumulative surveillance area over time without (gray) and with (red) IAA-94. Dashed line shows extrapolation of IAA-94–treated cells to reach the same surveillance area as control cells after 15 min. (**G**) Surveillance rate (left) and surveillance territory (right) (*n* = 22). (**H** and **I**) Effects of CLIC1 blockers on surveillance index [(H); *n* = 18, 22, and 22] and motility index [(I); *n* = 18, 17, and 21]. (**J**) Sholl analysis–derived total process length (left; *n* = 17, 17, and 16) and number of processes (right; *n* = 15, 23, and 21). (**K**) Schematic illustrating application regimes for (L) to (N). Top: Extracellular application via puff pipette. Bottom: Intracellular application by patch-clamping of microglia. (**L**) Left: Images of patch-filled microglia without and with IAA-94. Right: Surveillance index, normalized to control (*n* = 3 each). (**M**) As for (L) but with intracellular application of anti-CLIC1 (tmCLIC1omab; *n* = 5) or immunoglobulin G1 (IgG1) control antibody (3.5 μg/ml each; *n* = 6). (**N**) As for (L) and (M) but with extracellular puff application of anti-CLIC1 or control antibody (*n* = 6 each). (**O**) Images showing chemotactic extensions of microglial processes toward a laser ablation. Note that images are from reduced 25-μm *z*-stacks. (**P**) Microglial chemotaxis expressed as decrease in clear area not covered by microglial processes over time and comparison of time constants of the decay (inset; *n* = 10 and 8). Data indicate means ± SEM. Dots on bars show individual cells or single chemotactic events (P). Data are from three mice per condition. *P* values from paired [(G) to (J)] and unpaired [(N) to (P)] Student’s *t* tests, Wilcoxon signed-rank test [(H); 50 μM IAA-94], and Welch’s *t* test [(L) and (M)].

The obtained Cl^−^ channel expression profile, together with the absence of Cl^−^ channel activity in the plasma membrane, suggests that the decrease in surveillance by DIDS and NPPB involves intracellular CLIC1, which is susceptible to both substances (*28*–*32*). To test this hypothesis, we characterized the effects of acute pharmacological CLIC1 blockade on microglial morphodynamics via live imaging as described above. Application of the most commonly used CLIC-specific inhibitor indanyloxyacetic acid-94 (IAA) (*28*, *33*–*36*) rapidly reduced surveillance in a dose-dependent manner, resulting in a less complex morphology by reducing the number of process intersections, total process length, and number of processes determined by Sholl analysis (Fig. 3, A, B, H, and J, and movie S3). In line with an effect on CLIC1, the decrease in surveillance by DIDS was much weaker in the presence of IAA (fig. S3, A and B). Because both the ramification and motility of microglial processes affect surveillance (*3*), we additionally analyzed the cell size–independent motility index (see Materials and Methods). This revealed a reduction in motility in response to IAA (Fig. 3I), implying that the decrease in surveillance by CLIC1 blockade results from both deramification and impaired dynamics of microglial processes. Together, these changes result in a greatly reduced surveillance rate by, and territory of, individual microglia without CLIC1 function and a delay of ~60% in achieving the same cumulative surveillance as that of control cells (Fig. 3, E to G). The effects of IAA were recapitulated by a structurally different CLIC1 blocker, anthracene-9-carboxylic acid (A9C) (*33*, *34*, *36*), which decreases microglial morphodynamics with a comparable time course and magnitude (Fig. 3, C, D, and H to J, and movie S4).

To exclude indirect effects mediated by other cells, we additionally applied IAA specifically to microglia by adding the substance to the intracellular solution when patch-clamping the cells (Fig. 3K). As seen with bath application, intracellular administration of IAA led to a marked retraction of microglial processes and slowed surveillance compared to patch-clamped cells without IAA, showing continuous process extensions and retractions (Fig. 3L). A similar decrease in surveillance was obtained when microglia were intracellularly perfused with an anti-CLIC1 antibody added to the patch solution (see Materials and Methods), as opposed to an isotype control, which had no effect. Notably, extracellular application of anti-CLIC1 at microglia via a puffing pipette left surveillance unaffected, consistent with an intracellular role of CLIC1 (Fig. 3, K to N). Because the antibody is also capable of inhibiting ion channel activity by binding to the very N-terminal region of CLIC1, which is located extracellularly in the case of its membrane-bound form (*37*), these findings support our electrophysiological data indicating that CLIC1 does not regulate surveillance by acting as a plasma membrane ion channel.

In addition to surveillance, microglia also exhibit highly targeted process movements, i.e., chemotaxis, for example, in response to an increase in extracellular ATP concentration, reflecting a danger signal. This effect was mimicked by laser ablation–induced focal tissue damage, generating robust microglial chemotaxis toward the ablation site within ~10 min. However, in contrast to surveillance, CLIC1 blockade had no effect on the extent or time course of chemotaxis (Fig. 3, O and P, and movies S5 and S6).

**Fig. 4.**
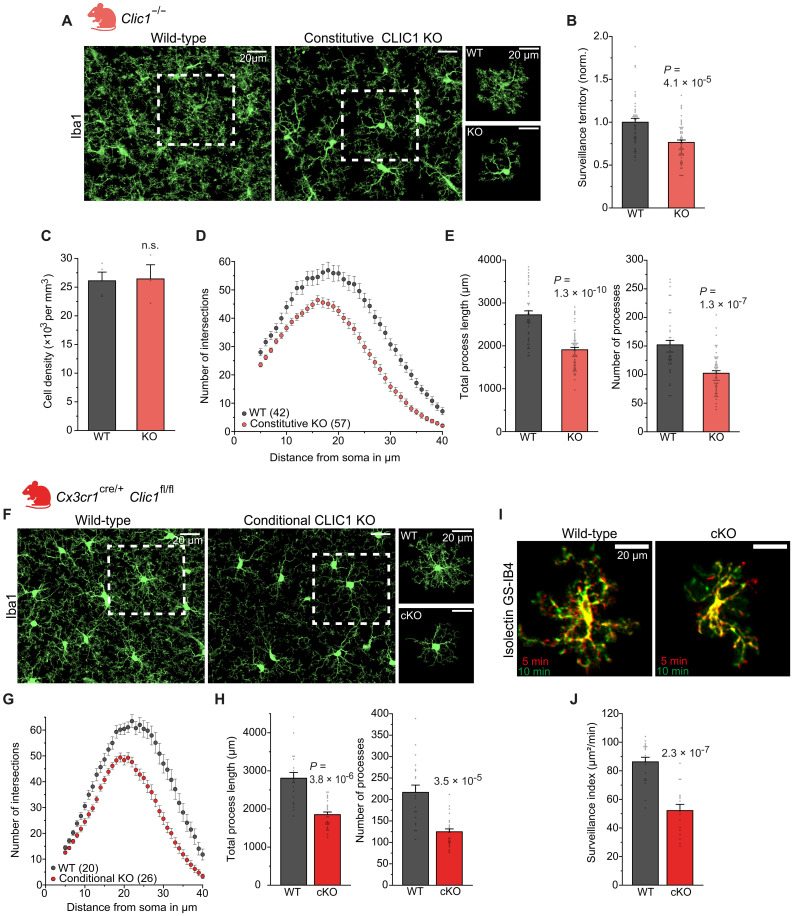
CLIC1-deficient microglia exhibit reduced ramification and surveillance. (**A**) Representative low-power images and close-up views showing Iba1 labeled cortical microglia in perfusion-fixed brain tissue from WT and constitutive CLIC1 KO mice (*Clic1*^−/−^). (**B**) Analysis of the surveillance territory of microglia in WT and CLIC1 KO mice (normalized to the WT; *n* = 42 and 57). (**C**) Analysis of microglial cell density between genotypes (*n* = 4 and 3). (**D**) Analysis of microglial ramification by Sholl analysis for microglia from WT and CLIC1 KO mice. (**E**) Comparison of the total process length (left) and the number of processes (right) derived from Sholl analysis between genotypes (*n* = 42 and 57). (**F** to **H**) As for (A), (D), and (E) but for microglia from conditional CLIC1 KO mice (cKO) (*Cx3cr1*^cre/+^ *Clic*^fl/fl^; *n* = 20 and 26). (**I**) Superimposed images of cortical microglia captured 5 min apart from WT and conditional CLIC1 KO mice, acutely labeled with Alexa Fluor 594–conjugated Isolectin GS-IB4 (colors as in Fig. 1A). (**J**) Quantification of surveillance averaged over the last 5 min for WT (*n* = 19) and conditional CLIC1 KO (*n* = 15) microglia. Data information: Data indicate means ± SEM. Dots on bars show number of cells and, in (C), number of mice. Data are from three mice per experimental condition. *P* values are from Mann-Whitney *U* test [(B) and (E), right], unpaired Student’s *t* tests [(C) and (J)], and Welch’s *t* test [(E), left, and (H)].

Consistent with these pharmacological interventions, microglia in CLIC1-deficient mice (*38*) presented significantly reduced complexity and length of their processes compared with those in wild-type (WT) littermates (Fig. 4, A, D, and E). Together with an unchanged cell density, this results in a significantly reduced surveillance territory (Fig. 4, B and C). No up-regulation of other *Clic* subtypes was observed in CLIC1 knockout (KO) mice (fig. S4, A and B), and application of IAA to CLIC1-deficient microglia caused only a minor but nonsignificant change in their morphology (fig. S4, C to E). Together with the transcriptomic data, this suggests that the effects of IAA on microglia are predominantly due to inhibition of CLIC1. In addition, we observed an increase in immunofluorescence of the lysosomal marker CD68 in microglia from CLIC1-deficient mice (fig. S4F).

Because CLIC1 is also expressed in endothelial cells (*28*, *38*), we additionally validated our findings in mice lacking CLIC1 only in microglia and other myeloid lineage cells (see Materials and Methods). Apart from a similarly reduced complexity of microglial processes in this model compared to constitutive gene KO (Fig. 4, F to H), we found that surveillance was also greatly reduced (Fig. 4, I and J), in line with acute pharmacological blockade. Together, these findings support a key role for CLIC1 in regulating microglial surveillance by affecting the length, ramification, and motility of cellular processes.

### CLIC1 control of microglial morphodynamics is independent of the chloride gradient but involves interactions with ERM proteins in actin-dependent processes

**Fig. 5.**
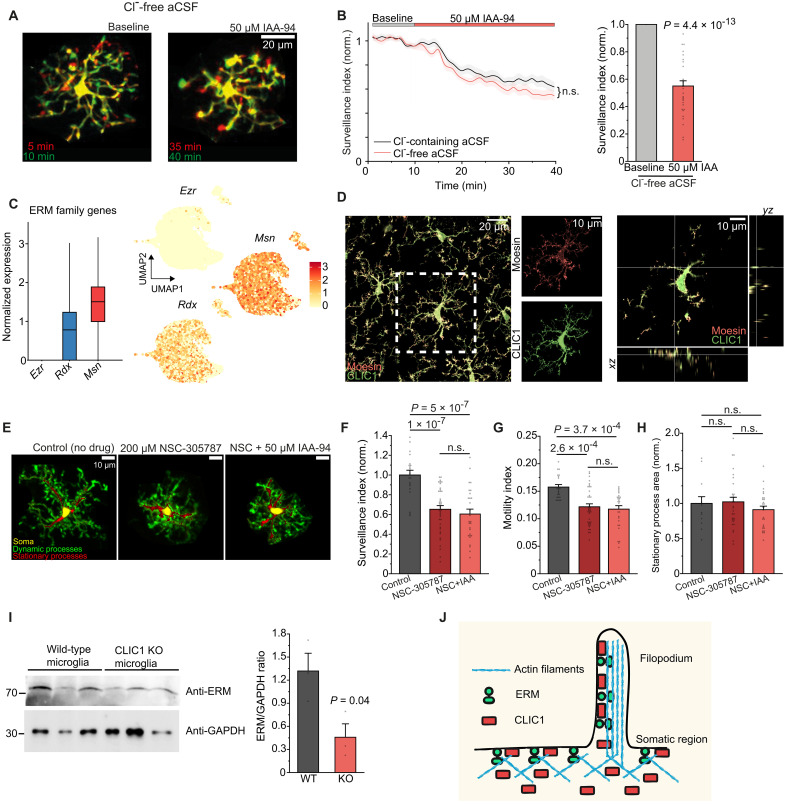
CLIC1 control of microglial morphodynamics is independent of Cl^−^ abundance and involves interactions with ERM proteins. (**A**) Images visualizing microglial surveillance under Cl^−^-free conditions before and with IAA-94. (**B**) Left: Time course of microglial surveillance with IAA-94 in Cl^−^-free aCSF. For comparison, IAA-94 effect in Cl^−^-containing aCSF from Fig. 3B is also shown. Right: Surveillance index in Cl^−^-free aCSF (*n* = 32). (**C**) Expression levels of ERM family genes in mouse microglia from pseudo-bulk data (left) and as UMAP from single cells (right). (**D**) Confocal images showing CLIC1 and moesin immunoreactivity in microglia. Left: Maximum projection and single-channel views. Right: Orthogonal projections of the highlighted cell. (**E**) Images of microglia exposed to ERM blocker NSC alone or in combination with IAA-94 for 45 min, showing somata, stationary, and dynamic processes. (**F** and **G**) Mean effects of ERM and CLIC1 blockers on (F) surveillance and (G) motility (*n* = 22, 39, and 28). (**H**) Mean changes in microglial area covered by stationary processes, normalized to control (*n* = 13, 31, and 27). (**I**) Left: Western blot showing the expression of ERM proteins and glyceraldehyde-3-phosphate dehydrogenase (GAPDH; loading control) in microglia from WT and CLIC1 KO mice. Right: Relative ERM expression, normalized to GAPDH expression (*n* = 3 each). (**J**) Schematic (created in BioRender: A. Rifat (2025); https://BioRender.com/sn2bgql) showing the presumed organization of CLIC1 and ERM proteins with the plasma membrane and actin cytoskeleton. Data indicate means ± SEM [except (C)]. Dots on bars show individual cells, except for (I), representing cells per hemisphere. Data for (B) are from 3 mice; for (F) to (H) from 7 (control), 5 (NSC), and 6 (NSC with IAA-94) mice, respectively; and for (I) from 4 mice. *P* values are from Mann-Whitney *U* test (B, left); unpaired (I) and paired (B, right) Student’s *t* test; one-way analysis of variance (ANOVA) with Tukey’s post hoc test [(F) and (H)]; and Kruskal-Wallis test with Dunn’s post hoc test (G).

The above findings suggest an intracellular role of CLIC1 in regulating microglial morphodynamics. To test whether the effects of CLIC1 rely on the abundance of Cl^−^, we exposed slices for 45 min to Cl^−^-free extracellular solution, leading to rapid disruption of the Cl^−^ gradient across the plasma membrane and, consequently, also to a reduction of the intracellular Cl^−^ concentration (*39*, *40*). To prevent any changes in neuronal activity, tetrodotoxin (TTX) was added throughout the experiment, which does not affect microglial surveillance (*1*). Notably, as with IAA, Cl^−^ replacement induced a retraction of microglial processes and a decrease in surveillance without affecting chemotaxis (fig. S5, A to D). However, in slices preequilibrated in Cl^−^-free extracellular solution for 45 min, IAA still reduced microglial surveillance and ramification, with effect sizes that did not differ from that obtained in the presence of Cl^−^ (Fig. 5, A and B; compare Fig. 3, B and H). Consistent with our electrophysiological data, these findings support an intracellular role for CLIC1 that is largely independent of cellular Cl^−^ homeostasis.

CLICs have been reported to interact with cytoskeletal components, specifically proteins of the ERM family, which are involved in linking the plasma membrane to the underneath cortical actin network in both somatic regions and filopodia (*41*, *42*). Our sequencing data revealed that microglia express high levels of moesin and, to a lesser extent, radixin (Fig. 5C). Immunofluorescence analysis of moesin revealed prominent labeling of the outer microglial membrane and colocalization with CLIC1 signal particularly in the widely branched processes (Fig. 5D). Conceivably, CLIC1 may regulate microglial surveillance through interactions with ERM proteins by affecting actin cytoskeletal organization. To test this hypothesis, we acutely blocked ERM proteins using the specific small molecule inhibitor NSC-305787 (NSC) (*43*). Application of NSC significantly decreased microglial surveillance by reducing both process ramification and motility (Fig. 5, E to G), achieving an effect size similar to that of CLIC1 blockade. To test for a mechanistic relationship between CLIC1 and ERM proteins, we coapplied IAA in the presence of NSC, which produced no additional effects compared with those of NSC alone (Fig. 5, E to G). In contrast, when actin dynamics were impaired via different mechanisms, such as the inhibition of Rac GTPase or the Arp2/3 complex, coapplication of IAA then elicited a further reduction in surveillance (fig. S5, E and F).

To gain further mechanistic insight, we examined potential close-range interactions between CLIC1 and moesin. To this end, we performed coimmunoprecipitation (Co-IP) experiments in human embryonic kidney (HEK) 293 cells recombinantly expressing CLIC1 and moesin, as well as in acutely isolated microglia from whole brain. Using CLIC1 as bait, we were unable to detect moesin, neither in its dormant nor active phosphomimetic forms (fig. S5, G and H) (*44*). Together with our immunofluorescence data, these findings suggest that, although CLIC1 and moesin are enriched in the same microglial compartment, they do not seem to physically interact. Given their converging function, this supports a mechanism in which CLIC1 acts upstream of moesin. In line with this, we observed reduced moesin protein expression in CLIC1-deficient microglia (Fig 5I).

Microglial processes differ in their structural and morphodynamic properties, comprising (i) thicker stationary microtubule-dependent first-order processes as part of the cell backbone and (ii) thinner, highly dynamic second-order processes containing filamentous actin (*11*). The decrease in surveillance and motility indices upon CLIC1 or ERM blockade represents a reduction in motile microglial area in absolute terms (surveillance) or relative to cell size (motility index) and mainly reflects the total changes in process extensions and retractions over time due to the immobility of somata. However, no conclusions can be drawn as to the extent to which normally stationary processes may also contribute to this decrease or, from a mechanistic point of view, whether their microtubules may also be subject to CLIC1 and ERM regulation. To address this, we determined the changes in total area of stationary microglial processes upon IAA and NSC application and compared it with that of untreated cells. Notably, neither CLIC1 blockade alone nor in combination with ERM blockade altered the stationary processes (Fig. 5, E and H), underscoring a specific role of CLIC1 in regulating actin-driven dynamics. Co-IP from whole brain–isolated microglia revealed β-actin in complex with CLIC1, demonstrating close physical association (fig. S5I). This finding is consistent with the predominant expression of β-actin transcripts in microglia compared to other brain cells (*45*), and with the protein localizing specifically to their ramified, motile processes (fig. S5, J and K). Together, these results suggest that the effects of CLIC1 on microglial morphodynamics are independent of Cl^−^ but involve nonphysical interactions with cytoskeletal ERM proteins, of which microglia mainly express moesin. CLIC1- and ERM-induced effects are functionally convergent and selectively affect actin-dependent second-order processes (Fig. 5J).

### CLIC1 regulates NLRP3-dependent IL-1β release

**Fig. 6.**
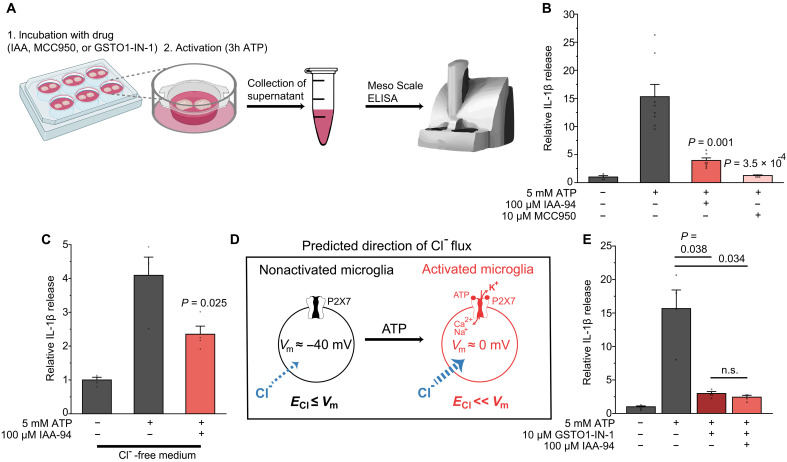
CLIC1 regulates NLRP3-dependent IL-1β release in response to purinergic microglial activation. (**A**) Scheme (created in BioRender: A. Rifat (2025); https://BioRender.com/jx63a07) illustrating the workflow for determining IL-1β levels from supernatants of brain slices upon purinergic stimulation (5 mM ATP, 3 hours) to simulate acute brain damage. (**B**) Mean effects on IL-1β release by inhibition of CLIC1 and NLRP3 with IAA-94 and MCC950, respectively (*n* = 4, 8, 7, and 4). (**C**) Mean effect on IL-1β release by inhibition of CLIC1 in Cl^−^-free extracellular medium (*n* = 4 each). (**D**) Schematic illustrating the predicted direction of Cl^−^ flux under the given experimental conditions for nonactivated and ATP/P2X7-activated microglia. Blue arrows indicate the direction of the electrochemical gradient for Cl^−^ per condition. *E*_Cl_, Cl^−^ equilibrium potential; *V*_m_, membrane potential. (**E**) Mean effect of GSTO1-IN-1 on IL-1β release, with no additional effect upon coapplication with IAA-94 (*n* = 6, 4, 4, and 4). Data information: Data indicate means ± SEM and are normalized to control condition. Dots on bars show number of slices. Data are from four mice per experimental condition. *P* values are from Welch’s *t* test (B), unpaired Student’s *t* tests (C), and Welch’s ANOVA with Games-Howell post hoc test (E). h, hours.

In peripheral macrophages, CLICs were shown to interact with components of the NLRP3 inflammasome (*36*, *46*), suggesting the involvement of CLIC1 in NLRP3-dependent IL-1β release from brain-resident microglia. To test whether CLIC1 contributes to microglial IL-1β release, we exposed brain slices to extracellular ATP to simulate acute brain damage (Fig. 6A) (*47*, *48*). While ATP potently induced P2X7-dependent IL-1β release (*47*), which was almost completely blocked by the NLRP3 antagonists MCC950 and oridonin, IL-1β levels were also substantially suppressed by >70% by CLIC1 blockade with IAA (Fig. 6B and fig. S6, A and B). Dual administration of MCC950 and IAA produced no additive effect compared with MCC alone (fig. S6B). To investigate the role of CLIC1 in mediating Cl^−^ efflux for NLRP3 activation (*36*), we performed the experiment with slices preequilibrated in Cl^−^-free extracellular medium (with TTX added). ATP-evoked IL-1β release was still significantly reduced by CLIC1 blockade under these conditions, although to a lesser extent than in the presence of Cl^−^ (Fig. 6C), suggesting a mechanism of CLIC1 that is largely independent of Cl^−^ efflux. This is supported by measurements of the microglial membrane potential during ATP exposure, which is ~0 mV due to the activation of nondesensitizing P2X7 receptors (*47*), resulting in an electrochemical Cl^−^ gradient that is directed inwardly under our conditions (Fig. 6D).

To further investigate how CLIC1 inhibition interferes with NLRP3 signaling, we explored whether this involves CLIC1’s oxidoreductase activity, given its structural and functional homology to omega-class glutathione *S*-transferase 1 (GSTO1) (*34*, *49*). To this end, we tested GSTO1 inhibitor 1 (GSTO1-IN-1, also known as C1-27), which covalently binds to Cys^32^ within the active site of GSTO1 (*50*). This residue is structurally homologous to Cys^24^ in CLIC1, the proposed catalytic site responsible for its oxidoreductase function (fig. S6C) (*34*, *49*, *51*). Notably, GSTO1-IN-1 robustly suppressed IL-1β release, and coapplication with the CLIC1 inhibitor IAA failed to produce an additive effect (Fig. 6E), suggesting that CLIC1 modulates NLRP3 activation via a GSTO1-like redox mechanism. We also tested whether the decrease in IL-1β release upon CLIC1 blockade may involve actin-binding ERM proteins, since actin-dependent processes can modulate NLRP3 function (*52*), but we only observed a nonsignificant trend toward lower IL-1β levels upon blockade of ERM proteins with NSC (fig. S6D).

Given its role in microglial morphodynamics, we hypothesized that CLIC1 may affect changes in microglial volume upon ATP-induced activation, as cell volume regulation is known to affect NLRP3 activation (*53*). Activated microglia adopted a deramified amoeboid-like shape accompanied by significant volume loss (fig. S6, E to G). However, CLIC1-inhibited microglia experienced much less volume change, mainly because of a significantly smaller cell volume prior to ATP exposure (fig. S6G). These findings suggest that CLIC1-dependent regulation of IL-1β release occurs via an NLRP3-dependent mechanism involving oxidoreductase activity.

### CLIC1-dependent properties in mice are recapitulated in human microglia

**Fig. 7.**
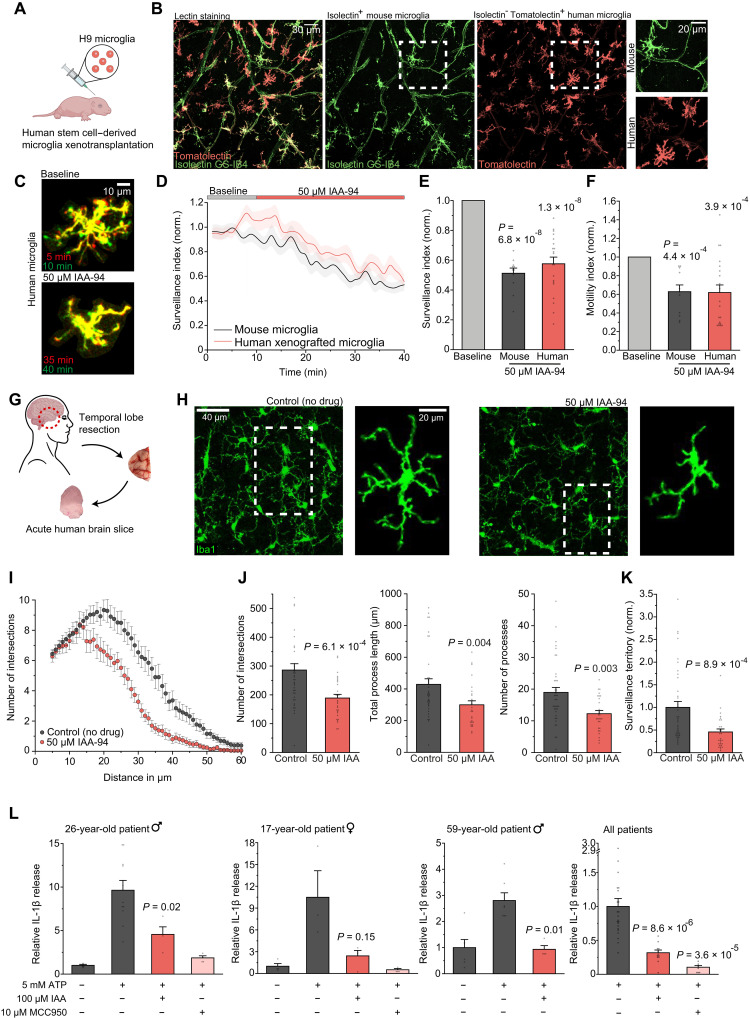
CLIC1-dependent properties in mice are recapitulated in xenografted human microglia and in human brain tissue. (**A**) Illustration of xenotransplantation model used in (B) to (F) (*54*). (**B**) Images of brain slices stained with Isolectin GS-IB4 and Tomatolectin to distinguish mouse (Isolectin^+^) from human (Tomatolectin^+^/Isolectin^−^) microglia (*98*). (**C**) Superimposed images before and with IAA-94. (**D**) Time course of surveillance before and with 50 μM IAA-94. (**E** and **F**) Effects of IAA-94 on (E) surveillance index and (F) motility index of human and murine microglia (*n* = 11 and 20). (**G**) Scheme depicting the utilization of acutely resected surgical tissue from human temporal lobe. (**H**) Representative images showing microglia in the human neocortex in the absence (control) and presence of 40-min exposure to IAA-94. (**I**) Comparison of microglial ramification without and with IAA-94. (**J**) Effects of IAA-94 (*n* = 39 and 29) on number of intersections (left), total process length (middle), and number of processes (right). (**K**) Effect of IAA-94 on the surveillance territory of microglia, normalized to control (*n* = 39 and 29). (**L**) Comparison of ATP-evoked IL-1β release in dependence of IAA-94 and MCC950 in three individual patients (left; patient 1: *n* = 4, 11, 4, and 4; patient 2: *n* = 4, 3, 4, and 4; patient 3: *n* = 6, 7, and 4) and pooled for all patients (very right; *n* = 22, 12, and 8). Individual data are normalized to control condition or to the ATP condition for pooled data. Data indicate means ± SEM. Dots on bars show number of cells [(E), (F), and (J)] or number of slices (L). Data are from three mice [(B) to (F)] and three individual patients [(H) to (L)]. *P* values are from paired [(E) and (F): murine microglia] and unpaired [(L): patients 1 and 3] Student’s *t* tests, Wilcoxon signed-rank test [(F): human microglia], Welch’s *t* test [(L): patient 2], and Mann-Whitney *U* tests [(J) to (L): combined data]. Illustrations are created in BioRender: A. Rifat (2025); https://BioRender.com/9mwwjbn and https://BioRender.com/8xo9x45.

As shown at the transcript level, *Clic1* is highly and nearly exclusively expressed in human brain microglia (see Fig. 2, G to K). To translate these findings to the human context, we used mice xenografted with stem cell–derived human microglia alongside their endogenous murine counterparts (*54*), enabling side-by-side comparison of human and mouse microglia within the same brain tissue (Fig. 7, A to F). Microglia were visualized in acute slices using fluorophore-coupled Isolectin GS-IB4 and Tomatolectin and were distinguished based on their different lectin-binding properties, with Tomatolectin-positive/Isolectin-negative cells representing human microglia and Isolectin-positive cells reflecting murine microglia (Fig. 7B). CLIC1 blockade by IAA reduced surveillance to a similar extent in both human and murine microglia, consistent with comparably high *Clic1* expression in both species.

We also repeated key experiments in brain slices obtained from acutely resected human temporal lobe (Fig. 7G; see Materials and Methods). As in mice, CLIC1 blockade by IAA resulted in a significantly less complex, deramified morphology of human brain microglia, as shown by a lower number of processes, intersections, and total process length, resulting in a significantly diminished surveillance territory (Fig. 7, H to K).

In addition, ATP-evoked release of IL-1β mediated by NLRP3 was greatly reduced by CLIC1 blockade in brain tissue of individual patients (Fig. 7L). Although there was considerable variability in basal and evoked IL-1β levels between patients, the effect sizes were comparable, reaching an average inhibition of IL-1β release of ~75% (Fig. 7L, far right).

## DISCUSSION

In this study, we identified CLIC1 as the most highly expressed Cl^−^ channel in mouse and human microglia and showed that it is essential for maintaining their physiological surveillance in the brain parenchyma. Mechanistic analyses identified that morphodynamic control by CLIC1 is independent of cellular Cl^−^ homeostasis and involves nonphysical interactions with ERM proteins, of which moesin is the dominant member in microglia. Consistent with an influence on the actin cytoskeleton, CLIC1 and ERM affected only actin-dependent microglial processes. We also found that inhibiting CLIC1 strongly suppressed the NLRP3-dependent release of IL-1β from mouse and human brain microglia.

Microglial surveillance depends on the resting membrane potential, which in nonactivated microglia is regulated by the tonic activity of THIK-1 K^+^ channels (*7*, *55*). Because the main counterion to K^+^ is Cl^−^, which affects the electrical membrane properties and morphology of many cells, we hypothesized that Cl^−^ channels may be involved in these processes in microglia. At first glance, the greatly reduced surveillance by the nonspecific Cl^−^ channel blockers DIDS and NPPB that we observed supports this assumption and is broadly in line with previous studies, mainly using primary cultures and cell lines, reporting a role for Cl^−^ channels in microglial morphology (*8*, *19*, *56*, *57*). However, our finding that the electrical membrane properties of cortical microglia are independent of extra- and intracellular Cl^−^ concentration provides strong evidence for the absence of constitutive Cl^−^ channel activity in the plasma membrane under physiological conditions, in contrast to previous findings in cultured microglia (*21*). Only when exposed to a hypotonic stimulus do microglia generate Cl^−^-dependent outward-rectifying currents, consistent with the activation of VRACs engaged in volume regulation (*16*). This implies that conductances other than Cl^−^ contribute to the rather positive resting membrane potential of ~−40 mV for nonactivated microglia and that, in contrast to other motile cells such as glioma (*58*), Cl^−^ channel activity is dispensable for the continuous movement of microglial processes through the narrow extracellular space. In light of this result, the effects of DIDS and NPPB on microglial surveillance suggest the involvement of a Cl^−^ channel–independent or intracellular target, which is corroborated by our transcriptome data revealing *Clic1* as the most highly expressed chloride channel in mouse and human brain microglia.

CLIC1, a member of the CLIC family comprising six highly conserved subtypes in vertebrates, is functionally distinct from a conventional ion channel and can exist in both soluble and membrane-associated forms (*42*), the latter being a prerequisite for acting as a conduit for anions. Mice with genetic deletion of CLIC1 are viable and fertile and appear essentially normal in an unstressed laboratory environment (*38*, *59*). In immune cells, CLIC1 has been studied mainly in peripheral macrophages, BV2 cells, and primary microglial cultures in the context of cell activation and immunological responses (*35*, *36*, *46*, *60*), but its physiological role in mouse and human brain microglia remained elusive. Given that both NPPB and DIDS may also target CLIC1 (*28*–*32*, *60*), we hypothesized that part of their effect on microglial morphodynamics was CLIC1-mediated. We found that abolishing CLIC1 using two different pharmacological inhibitors led to a greatly reduced surveillance of microglia, which was due to a reduction in the ramification, length, and motility of their processes. In support of our hypothesis, we determined a much weaker DIDS-mediated decrease in surveillance with concomitant specific inhibition of CLIC1 by IAA. Considering potential pharmacological off-target effects, we validated our findings in two independent genetic models with constitutive and microglia-specific deletion of CLIC1, both of which showed similar alterations in morphology and dynamic behavior as seen with IAA. Mechanistically, we found that the morphodynamic effects of CLIC1 were independent of extracellular Cl^−^ concentration, which is expected to also perturb intracellular Cl^−^ homeostasis (*40*, *61*, *62*). This suggests a role for CLIC1 that is also unlikely to be associated with Cl^−^ channel activity in intracellular membranes. In general, whether CLICs operate as classical ion channels under physiological conditions is still unresolved (*42*). A functional mode of CLIC1 as a membrane-bound ion channel has been reported in pathological contexts, such as in cancer cells and in activated microglia or macrophages exposed to oxidative stress or phagocytic challenge (*41*, *63*). Notably, transition of CLIC1 from its soluble to the membrane-associated form is redox-controlled, requiring an oxidizing cellular environment and is further promoted by acidic pH and high levels of cAMP (*37*, *49*), which do not apply to nonactivated microglia under physiological conditions.

If not as an ion channel, how could CLIC1 regulate microglial morphodynamics? Ultimately, microglial motility depends on fine-tuned membrane mechanics and the integrity of the complex molecular machinery steering the permanent remodeling of actin networks (*9*). This is supported by studies showing that interference with these mechanisms, for example, by deletion or blockade of the actin-interacting proteins Iba1, RhoA, or Arp2/3, leads to impairment of microglial morphology and motility (*10*, *64*, *65*) or to complete arrest of process movement when actin polymerization is blocked (*8*, *11*, *64*). CLICs, including CLIC1, are involved in manifold interactions with cytoskeletal components, such as actin-dependent membrane remodeling, integrin signaling, matrix adhesion, and endosomal trafficking (*22*, *66*, *67*). CLIC1 can directly interact with filamentous actin and is enriched in cellular extensions like filopodia and promotes their formation (*38*, *67*, *68*), which is associated with increased cell motility and migration (*69*–*71*). This is supported by our findings demonstrating enrichment of CLIC1 transcript and protein in microglial processes, along with the physical association of CLIC1 with β-actin. In addition, CLICs can bridge the plasma membrane to the underneath actin network, either directly or via interactions with ERM proteins, thereby affecting membrane-to-cell cortex attachment (*22*, *41*, *72*). We found that inhibiting ERM proteins phenocopied and completely occluded CLIC1-induced effects on microglial morphodynamics, consistent with expression of radixin and especially moesin in microglia, suggesting a mechanistic interaction between CLIC1 and ERM proteins.

How could inhibiting ERM proteins lead to reduced surveillance? Through their role as molecular linkers, ERM complexes regulate the organization and remodeling of the actin cytoskeleton and plasma membrane tension, thereby maintaining integrity of the cell cortex and regulating cell morphology and motility (*73*–*75*). In primary microglia, impairment of ERM function caused a decrease in membrane tension and internal osmotic pressure, resulting in alterations in cell morphology and membrane dynamics (*76*, *77*). Notably, ERM proteins, similar to CLIC1, are concentrated in filopodial extensions and promote their formation and structural integrity through lateral binding of actin bundles to the plasma membrane (*78*–*80*). This aligns well with our observations in brain-resident microglia, where moesin prominently localizes to the outer cell membrane and colocalizes with CLIC1. Likewise, moesin is found in dynamic filopodia in macrophages (*81*) and is involved in the reorganization of the actin cytoskeleton in primary microglia (*76*). Conceivably, blockade of CLIC1 or moesin, through detachment of actin bundles from the plasma membrane, would lead to destabilization and eventual collapse of microglial processes, as seen in a reduction in process ramification (compare schematic, Fig. 5J). Similarly, the constant actin remodeling that drives the motility of microglial processes is expected to be slowed if their rigidity and attachment sites for actin treadmilling are compromised (*81*). Consistent with this, we found that CLIC1 and ERM blockade resulted in retraction and slowed motility of actin-dependent second-order processes, whereas microtubule-dependent primary processes as part of the static backbone of microglia (*11*) remained unaffected. Unlike with β-actin, we did not obtain evidence for a direct interaction between CLIC1 and moesin. However, their colocalization and functional convergence support a model in which CLIC1 acts upstream of moesin. Elucidating the detailed structural and mechanistic basis of this relationship warrants further investigation.

In contrast to surveillance, targeted movement of microglial processes toward a chemotactic stimulus was unaffected by CLIC1 blockade, highlighting different underlying mechanisms. This is reminiscent of other motility-regulating determinants in microglia, such as THIK-1, which also controls surveillance but not chemotaxis (*7*), and may be related to the fact that the formation of new, e.g., chemotactic, filopodia can occur independently of an existing actin network (*82*), e.g., involved in surveillance. Similarly, blockade of the master actin nucleation complex Arp2/3 is critical for random cell motility but is dispensable for chemotaxis (*83*), analogous to the dependence of microglial surveillance but not chemotaxis on Arp2/3 (*64*). Another interesting finding of our study is that deprivation of extracellular Cl^−^ induces changes in microglial morphodynamics similar to those of CLIC1 or ERM blockade, i.e., a decrease in ramification and surveillance. Because our results do not support a Cl^−^ channel activity of CLIC1, and the effects of Cl^−^ depletion and CLIC1 are additive, this may be due to a reduction in membrane tension as a common consequence of both CLIC1 or ERM blockade and Cl^−^ depletion (*72*, *74*, *84*), thereby affecting the mechanobiology of the cell surface (*75*). As with CLIC1 blockade, microglial chemotaxis was unaffected when omitting extracellular Cl^−^, which contrasts with previous findings (*8*) but is consistent with recent data showing intact chemotaxis even in the absence of VRAC-dependent microglial volume control (*17*). Despite growing knowledge, mainly from macrophages and primary microglia, the precise mechanisms underlying the unique morphodynamic properties of central nervous system (CNS)–resident microglia remain poorly understood at the molecular level and warrant further investigation.

Another key finding of our study is the regulation of the NLRP3 inflammasome by CLIC1, the blockade of which reduced IL-1β release in mouse and human brain tissue by >70%. Notably, NLRP3 activation is regulated by numerous factors that are also influenced by CLIC1, such as actin polymerization, reactive oxygen species, or intracellular organelle function (*42*, *52*). To elucidate the mechanism, we tested the role of CLIC1 in mediating Cl^−^ efflux, which has been suggested to contribute to NLRP3 activation in peripheral macrophages via translocation of CLICs to the plasma membrane (*36*, *46*, *85*). However, our finding that CLIC1 blockade, albeit with somewhat reduced potency, still inhibited IL-1β release in Cl^−^-free extracellular medium, implies that putative Cl^−^ efflux via CLIC1 is unlikely to play a major role. Moreover, even with extracellular Cl^−^ present, the electrochemical gradient for Cl^−^ is always inward under our experimental conditions using P2X7 receptor activation as one of the most potent inducers of NLRP3. This is because P2X7 depolarizes microglia to ~0 mV in the continued presence of ATP (*47*), which is considerably more positive than the Cl^−^ equilibrium potential [which is ~−45 mV in adult cortical microglia; (*20*)], thus preventing Cl^−^ efflux via Cl^−^-permeable ion channels. Related to CLIC1’s structural homology to GSTO1, we found that inhibiting GSTO1 greatly suppressed IL-1β release. Simultaneous blockade of CLIC1 produced no additional effect, suggesting that CLIC1 regulates NLRP3 activation due to its GSTO1-like redox activity (*34*, *49*, *51*, *86*). In line with this, GSTO1 has been shown to regulate ASC oligomerization and NEK7 function, both of which are crucial components for NLRP3 activation (*87*, *88*). Moreover, CLICs, including CLIC1, have been reported to control ASC oligomerization in peripheral macrophages (*36*, *46*).

Our findings, particularly those relating to the control of proinflammatory cytokine release in the human brain, could have important clinical implications, as NLRP3 is a major driver of microglial-dependent inflammation affecting many CNS pathologies (*89*). Although peripheral human macrophages also show NLRP3-independent IL-1β release in response to ATP (*90*), our current and previous findings (*47*, *48*) demonstrate that ATP-evoked IL-1β release in mouse and human brain microglia is entirely dependent on NLRP3, highlighting its pathological role in the brain. Microglia play a central role in virtually all neurological diseases, profoundly influencing disease progression and outcome. Similar to the mechanisms described here for CLIC1, suppression of NLRP3 signaling via microglia-specific THIK-1 potassium channels is currently being investigated in clinical trials as a potential therapeutic approach for neurodegenerative diseases (*47*). Chronic inflammatory processes are also increasingly recognized as a major pathogenic factor in neuropsychiatric disorders. Notably, CLIC1 has recently been identified as a genetic risk locus in bipolar disorder, autism spectrum disorder, schizophrenia, depression, and posttraumatic stress disorder (*91*–*94*). Given its relevance for inflammatory regulation and disease genetics, CLIC1 may represent a promising therapeutic target across a wide spectrum of neurodegenerative and neuropsychiatric diseases. In support of this, CLIC1 displays remarkably high cell-type specificity, with almost exclusive expression in microglia in the human brain. Together, these findings underscore CLIC1 as a highly selective and translationally relevant target for modulating microglial activation in CNS disease.

## MATERIALS AND METHODS

### Compounds

IAA-94 (I117, Sigma-Aldrich), NSC-305787 hydrochloride (HY-18931A, MedChemExpress), NSC-23766 hydrochloride (HY-15723A, MedChemExpress), A9C (10449010, Thermo Fisher Scientific), DIDS (4523, Tocris), NPPB (0593, Tocris), 2-fluoro-*N*-[2-(2-methyl-1H-indol-3-yl)ethyl]-benzamide (CK-666; SML0006, Sigma-Aldrich), oridonin (09639, Sigma-Aldrich), GSTO1-IN-1 (HY-111530, MedChemExpress), and MCC950 (5479, Tocris) were dissolved and stored according to the manufacturer’s instructions. Drugs in electrophysiological and imaging experiments were applied via bath perfusion or added to the intracellular solution (in the case of IAA-94). Function-blocking anti-CLIC1 antibody (tmCLIC1omab) (*95*) was provided by M. Mazzanti, University of Milan, Italy, and was dissolved at 3.5 μg/ml in intracellular or extracellular solution. This antibody targets the N-terminal 20 amino acids of CLIC1, including the QWELF sequence, and is thereby also capable of recognizing the membrane-associated form and inhibiting its ionic current. As an isotype control, mouse immunoglobulin G1 (IgG1) was used (02-6100, Thermo Fisher Scientific).

### Human brain tissue

Human neocortical tissue of the medial temporal gyrus was obtained from three patients (one female and two males, age range of 17 to 59 years) diagnosed with drug-resistant epilepsy and who underwent temporal lobe resection, without further considering the individual’s patient history. Only tissue that had been resected to gain surgical access was used, avoiding the assumed disease focus in the hippocampus. All donors gave full written consent for the use of their material and clinical information in accordance with the World Medical Association’s Declaration of Helsinki for Medical Research. All experimental procedures complied with ethical requirements and were approved by the local ethics committee under license number EA2/111/14.

### Animals

For multiphoton imaging, patch-clamp, and RNA sequencing experiments, *Cx3cr1*^eGFP/+^ mice (*24*), which express enhanced green fluorescent protein (eGFP) under the control of the endogenous *Cx3cr1* locus, were used. For cytokine experiments, C57BL/6J mice were used. Myeloid cell–specific *Clic1*^−/−^ mice were generated by embryo transfer using sperm from *Clic1*-flox mice (strain no T013145, GemPharmatech) and *Cx3cr1*^cre/+^ transgenic females (*96*). Constitutive *Clic1*^−/−^ mice (*38*) were provided by H. Singh, The Ohio State University, USA. Transgenic *Rag2*^−/−^ *Il2r*γ^−/−^ *hCsf1*^KI^ mice xenotransplanted (at postnatal day 4) with stem cell–derived human microglia of H9 genetic background were provided by B. De Strooper (*54*). All experiments were performed using adult mice of both sexes, aged 3 to 6 months, which were housed in individually ventilated cages. All procedures involving the handling of live animals adhered to German animal welfare laws and were approved by the local health and social authorities (LaGeSo T0045/15, T0003/23, and T0041/22), as well as by the Ethical Committee for Animal Experimentation of KU Leuven (LA1210579, ECD P125/2022, and P132/2022) in accordance with Belgian regulations.

### Preparation of mouse brain slices

Mice were euthanized via decapitation under isoflurane anesthesia. Acute brain slices of 300-μm thickness of the neocortex (coronal plane) or hippocampus (horizontal plane) were prepared on a Leica VT1200 S microtome under protective conditions, using 4°C cold cutting solution containing 93 mM choline chloride, 20 mM Hepes, 30 mM NaHCO_3_, 2.5 mM KCl, 1.25 mM NaH_2_PO_4_, 10 mM MgCl_2_, 0.5 mM CaCl_2_, 25 mM glucose, 5 mM ascorbic acid, 3 mM Na-pyruvate, and 1 mM kynurenic acid, set to pH 7.4 and 310 to 315 mOsmol/liter. Slices were allowed to recover in warm cutting solution at 34° to 36°C for 12 to 15 min before being transferred to storage solution at room temperature, which contained 92 mM NaCl, 20 mM Hepes, 30 mM NaHCO_3_, 2.5 mM KCl, 1.25 mM NaH_2_PO_4_, 1 mM MgCl_2_, 2 mM CaCl_2_, 25 mM glucose, 5 mM ascorbic acid, 3 mM Na-pyruvate, and 1 mM kynurenic acid, which were set to pH 7.4 and 300 mOsmol/liter, until experimental use. Solutions were equilibrated with 95% O_2_/5% CO_2_.

Using acute brain slices represents a compromise that allows microglia to be studied close to their natural brain environment while enabling mechanistic pharmacological and electrophysiological investigations under highly controlled conditions, which is not feasible in vivo. However, due to unavoidable tissue damage caused by slicing, gradual spatiotemporal morphological changes in microglia occur, especially near the slice surface. To minimize such effects, we used protective slicing solution, imaged and recorded microglia located at depths >80 μm below the slice surface, and performed experiments within 4 hours of slicing.

### Preparation of human brain slices

As previously described (*47*), resected tissue from the human temporal lobe was rapidly transferred from the operating theater to the laboratory within 10 to 15 min in 4°C cold sterile cutting solution containing the following: 87 mM NaCl, 2.5 mM KCl, 3 mM MgCl_2_, 0.5 mM CaCl_2_, 10 mM glucose, 75 mM sucrose, 1.25 mM NaH_2_PO_4_, and 25 mM NaHCO_3_, set to pH 7.4 and 310 to 315 mOsm/liter, equilibrated with 95% O_2_/5% CO_2_. Acute coronal brain slices 300-μm thick were immediately prepared in ice-cold oxygenated cutting solution. Slices were allowed to recover for 30 min at 34° to 36°C in the same solution in which they were kept at room temperature until experimental use. Pharmacological treatment was administered to the slices for a duration of 40 min, after which they were fixed and further processed for immunohistochemical staining.

### Microglia isolation

Microglia were isolated from adult mouse brains (excluding the cerebellum and olfactory bulbs) using two distinct methods. For fluorescence-activated cell sorting (FACS)–based isolation, we used a previously described protocol (*97*), processing brains from *Cx3cr1*^eGFP/+^ mice and performing all steps at 4°C to prevent activation-related transcriptional changes. Briefly, cells were mechanically dissociated in Hibernate-A medium via a 1-ml Dounce homogenizer with a loose pestle. Cells were then filtered through a 70-μm cell strainer. Homogenates were centrifuged at 400 rpm for 6 min in a swing bucket rotor centrifuge. Myelin and debris layer were removed after the cells were resuspended in isotonic Percoll solution, followed by centrifugation at 3000 rpm for 10 min (without engagement of the brake). Cells were washed and centrifuged at 400 rpm for 10 min. GFP-positive microglia were isolated via FACS with a BD FACSAria III sorter. Nonviable cells were excluded on the basis of 4′,6-diamidino-2-phenylindole staining. Cells were counted via a hemocytometer.

For magnetic-activated cell sorting (MACS)–based isolation, brains were enzymatically and mechanically dissociated using the Adult Brain Dissociation Kit and the gentleMACS Octo Dissociator with Heaters (Miltenyi Biotec), according to the manufacturer’s protocol. Briefly, following tissue dissociation and debris removal, microglia were magnetically labeled with CD11b MicroBeads and enriched by positive selection using LS columns. Cells were washed and counted by hemocytometry.

### Single-cell RNA sequencing

FACS-isolated microglia were processed on a Chromium Single-Cell Platform via a Chromium Next GEM Single-Cell 3′ GEM Library and Gel Bead Kit (v.3 chemistry, 10x Genomics) and a Chromium Next GEM Chip G Kit according to the manufacturer’s instructions. Sequencing of the generated libraries was performed on an Illumina NovaSeq 6000 system. The data were processed with cellranger count (version 5.0.0) using mouse mm10-2020-A genome. The downstream analysis was carried out with Seurat (version 5.0.0). Cells with fewer than 200 genes and genes expressed in fewer than three cells were excluded from the analysis. We further filtered out cells with unique feature counts over 5000 or fewer than 200, as well as cells with more than 20% mitochondrial counts. Normalization was performed using the NormalizeData() function, applying the “LogNormalize” method with a scale factor of 10,000. Highly variable features were identified using the FindVariableFeatures() function with the variance stabilizing transformation (vst) method, selecting the top 2000 variable genes. Genes were scaled and subjected to principal components analysis using the top 2000 variable genes. Clustering was carried out by constructing an SNN graph using the first 10 principal components. Clustering resolution was set to 0.5. Uniform manifold approximation and projection (UMAP) dimensionality reduction was applied to visualize the clusters in a two-dimensional space using the RunUMAP() function, with the first 10 principal components as input. To validate our results in the human context, RNA sequencing data from myeloid cells isolated from the human brain (*26*) were analyzed following the same routines.

### Extra- and intracellular solutions

For live imaging and electrophysiological experiments, brain slices were perfused with bicarbonate-buffered aCSF at 34° to 36°C containing the following: 125 mM NaCl, 2.5 mM KCl, 25 mM NaHCO_3_, 1.25 mM NaH_2_PO_4_, 2 mM CaCl_2_, 1 mM MgCl_2_, and 10 mM glucose, set to pH 7.4 and 300 mOsm/liter, equilibrated with 95% O_2_/5% CO_2_. For depletion of extracellular chloride, NaCl, KCl, CaCl_2_, and MgCl_2_ were substituted with the respective gluconate (Na^+^, K^+^, Ca^2+^)– or sulfate (Mg^2+^)–based salts. For experiments using NSC, a Hepes-buffered aCSF containing 92 mM NaCl, 20 mM Hepes, 30 mM NaHCO_3_, 2.5 mM KCl, 2 mM CaCl_2_, 1 mM MgCl_2_, 1.2 mM NaH_2_PO_4_, and 25 mM glucose, set to pH 7.4 and 300 mOsm/liter, equilibrated with 100% O_2_, was used. To prevent changes in neuronal activity, 0.5 μM TTX was added to all extracellular solutions. When stock solutions of substances were prepared in dimethyl sulfoxide (DMSO), equivalent amounts of DMSO were added to the control solutions (maximum of 0.1%).

For electrophysiological experiments, microglia were whole-cell patch-clamped with an intracellular solution containing the following: 130 mM KCl or 140 mM K-gluconate (stated in the respective figures), 4 mM NaCl, 1 mM CaCl_2_, 1 mM MgCl_2_, 10 mM Hepes, 10 mM EGTA, 4 mM MgATP, and 0.5 mM Na_2_GTP, set at pH 7.2 and 290 ± 5 mOsm/liter. For patch-clamp experiments in which microglia were perfused with substances via the patch pipette and subsequently live imaged, EGTA and CaCl_2_ were omitted from the intracellular solution, and changes in the osmolarity were adjusted.

### Electrophysiological recordings of microglia

Patch pipettes for whole-cell recordings of microglia were pulled from borosilicate glass, yielding a tip resistance of 4 to 5 megohm. Signals were recorded with an Axopatch 200B amplifier, filtered at 1 kHz (10 kHz for the membrane test) and digitized at 10 kHz via a Digidata 1440 (both from Molecular Devices). Data acquisition and offline analysis were performed via pClamp 10 software. Electrode junction potentials were compensated. For recordings using Cl^−^-free extracellular solution, an agar salt bridge containing 3 M KCl dissolved in 4% agar in H_2_O (w/v) was used to provide an electrical connection between the Ag/AgCl reference electrode and the bath and to prevent shifts in liquid junction potential when changing Cl^−^ concentrations. The resting membrane potential (*V*_m_) was determined in current-clamp mode (*I* = 0) immediately after the whole-cell configuration was established. Input and series resistances were analyzed from voltage clamped cells held at −30 mV by applying 10-mV hyperpolarizing voltage steps. Current-voltage (*I*-*V*) relationships were obtained from current responses to 80-ms voltage steps at a holding potential of −30 mV.

### Immunohistochemistry

WT and CLIC1 transgenic mice were sacrificed by isoflurane overdose and immediately fixed by transcardial perfusion with 4% paraformaldehyde (PFA) in 0.1 M phosphate-buffered saline (PBS; 0.9% NaCl). Brains were then removed, and hemispheres were subjected to postfixation in 4% PFA at 4°C overnight. Fixed hemispheres were cryoprotected in PBS containing 30% sucrose for 48 hours and then sectioned at 16- or 100-μm thickness on a cryostat. Human brain slices were immersion-fixed in 4% PFA (in PBS) at 4°C overnight.

Slices were blocked in PBS containing 10% normal goat serum (NGS) and 0.5% Triton X (0.1% for CLIC1 staining) for 1 hour, followed by incubation with primary antibodies in PBS containing 5% NGS and 0.3% Triton X (0.1% for CLIC1) at 4°C for 48 to 72 hours. Microglia were immunolabeled with guinea pig anti-Iba1 (234308, 1:500, Synaptic Systems) and/or rat anti-CD68 (ab53444, 1:500, Abcam) primary antibodies. CLIC1 and moesin were stained using rabbit anti-CLIC1 (D7D6H, #53424, 1:500, Cell Signaling) and rabbit anti-moesin (ab52490, 1:500, Abcam).

Slices were then washed with PBS and incubated overnight with secondary antibodies in PBS containing 3% NGS and 0.1% Triton X (0.05% for CLIC1), with goat anti-guinea pig Alexa Fluor 488 (A-21450, 1:500, Thermo Fisher Scientific), goat anti-guinea pig Alexa Fluor 568 (A-11075, 1:500, Thermo Fisher Scientific), goat anti-rabbit Alexa Fluor 568 (A-11036, 1:500, Thermo Fisher Scientific), goat anti-rabbit Alexa Fluor 647 (A-21245, 1:500, Thermo Fisher Scientific), and/or goat anti-rat Alexa Fluor 647 (A-48265, 1:500, Thermo Fisher Scientific). Slices were mounted on glass slides using mounting medium and stored at 4°C.

To avoid potential cross-reactivity between secondary antibodies and nontarget primary antibodies during costainings, CLIC1 immunolabeling was performed first. Only after completion of CLIC1 immunolabeling were additional markers (Iba1 and moesin) stained. For costaining with rabbit anti-moesin, the primary antibody was directly fluorescently conjugated using the FlexAble 2.0 CoraLite Plus 488 Kit (KFA501, Proteintech) prior to application.

### RNAscope in situ hybridization

RNAscope Multiplex Fluorescent Reagent Kit v2 (Advanced Cell Diagnostics) was used on 16-μm cryosections from PFA-fixed mouse brains (WT and constitutive CLIC1 KO). Mouse-specific probes targeting *Clic1*, *Clic4*, and *Clic5* were hybridized according to the manufacturer’s protocol, followed by tyramide–based signal amplification. Sections were costained with guinea pig anti-Iba1 (234308, 1:200, Synaptic Systems) and fluorophore-conjugated secondary antibody. Slides were mounted using ProLong Gold and imaged by confocal microscopy.

### Two-photon and confocal imaging

Microglia in acute brain slices were live imaged at a depth of 80 to 150 μm below the slice surface using a Nikon A1R+ multiphoton microscope [with a 25× lens, numerical aperture (NA) 1.1] and a Spectra-Physics laser (Mai Tai Insight DeepSee Dual). For time-lapse imaging of microglial surveillance, 50-μm stacks with 2-μm *z*-intervals were acquired every minute using the laser tuned to a wavelength of 920 nm at a pixel dwell time of 2.2 μs. Images of fixed slices were taken on a Nikon A1R-si+ scanning confocal microscope (with a 40× lens, NA 1.3). Images typically had a resolution of 512 by 512 (live imaging) or 1024 by 1024 pixels (fixed tissue) covering a square field of view of 258 μm. Directed process motility (chemotaxis) was induced by ablating a small 5- to 10-μm volume of tissue by briefly increasing the laser power to 95% for 3 to 5 s. Image stacks of 25-μm thickness at 2-μm *z*-intervals were acquired every 30 s, with the laser ablation site placed in the center of the field of view by ensuring that adjacent microglia were not directly damaged.

Live imaging of microglia was based on their genetically encoded GFP label (*Cx3cr1*^eGFP/+^ mice) or on acute labeling with fluorophore-coupled lectin dyes (for *Clic1*^−/−^ and xenografted mice). For the latter, slices were incubated in darkness at room temperature in oxygenated extracellular solution containing Alexa Fluor 488–conjugated Isolectin GS-IB4 (25 μg/ml; I21411, Thermo Fisher Scientific) and DyLight 594–conjugated *Lycopersicon esculentum* (Tomato) Lectin (25 μg/ml; DL-1177-1, Vector Laboratories) for 30 min. Human microglia were identified based on their Tomatolectin-positive/Isolectin-negative staining (*98*), while murine microglia were positive for both lectins. For slices from *Clic1*^−/−^ mice, Alexa Fluor 594–conjugated Isolectin GS-IB4 (I21413, Thermo Fisher Scientific) was used to label microglia.

### Analysis of imaging data

For analysis of microglial surveillance and chemotaxis, multiphoton images were processed in ImageJ and MATLAB as previously described (*7*) using custom-written code (available at https://github.com/AttwellLab/Microglia). Briefly, after background subtraction, median filtering and correction of four-dimensional (4D) hyperstacks for lateral and *z*-drift, individual microglia within the entire image stack were manually selected and binarized. Calculation of added or removed pixels [i.e., process extensions (PE) and process retractions (PR)] frame by frame results in the surveillance index, defined as the sum of nonzero pixels in both PE and PR, normalized to the average of the first 10 min of the experiment (baseline, no drug). This index is a measure of the brain volume surveilled by microglia over a given time, considering the rate of process movement as well as the total number and length of processes. For a typical experiment, the surveillance index was determined as the mean index in the drug between 35 and 40 min, compared with the mean of the baseline (minutes 1 to 10, internal control). The cell size–independent motility index was determined by normalizing the surveillance index to the mean cell area per time, reflecting only the dynamic parts of microglia, i.e., mainly their motile processes. The area comprising nonmotile processes was analyzed from minimum intensity *z*-projections of the 4D images (omitting the somata) over a period of 15 min for the respective conditions. Microglial surveillance territories were determined from 3D image stacks by analyzing the convex hull area of individual microglia, which reflects the smallest convex polygon encompassing all distal process tips.

Chemotaxis was determined by dividing the processed images into radial sections in which the area enclosed by the front of chemotactic microglial processes was calculated for each time point. This results in a decrease in the “clear area” as chemotaxis progresses until a minimum is reached when all processes have reached the ablation site. Time constants (tau) were calculated via monoexponential fitting of the “clear area” decay.

Microglial morphology was determined by Sholl analysis, as previously described (*7*). Briefly, raw images were preprocessed in ImageJ as described above, and individual microglia were binarized and analyzed in 3D. For live imaging experiments, the control and drug conditions were determined from image stacks acquired at minute 5 during baseline (internal control) and minute 35 during drug exposure. In other experiments comparing different treatments or genotypes, slices were exposed to the drug(s) for 45 min (or no treatment for the control) in aCSF at 34° to 36°C and imaged either immediately afterward or following fixation and immunolabeling of microglia. Microglia reconstructions were performed using automated 3D cell tracing in Vaa3D software (https://home.penglab.com/proj/vaa3d/home/index.html). Morphological Sholl parameters (number of intersections and processes) were extracted from the obtained 3D cell skeletons using custom code written in MATLAB. Determination of the total length of processes was calculated as the sum of the Euclidean distances between consecutive nodes along the reconstructed skeleton of the cell.

For quantification of mRNA abundance and distribution within individual microglia, confocal image stacks of RNAscope-labeled sections were analyzed in Imaris (Bitplane) using the Surface module. Based on the Iba1 immunofluorescence signal, individual microglia were binarized in 3D. Within these cells, the mRNA signal (*Clic1*, *Clic4*, or *Clic5*) was binarized and rendered as separate surfaces. Somata and processes were defined as distinct Iba1 subvolumes, allowing assignment of mRNA signal to either compartment. The mRNA volume was quantified separately in processes and somata, enabling calculation of a process-to-soma mRNA ratio per cell. Total mRNA volume was further normalized to the respective cell volume. All imaging analyses were performed with the experimenter blinded to the experimental conditions or genotypes.

### ELISA measurements of IL-1β release

As previously described (*7*, *47*), 300-μm-thick brain slices were prepared under sterile conditions using cold oxygenated Hepes-buffered solution (Minimum Essential Medium (MEM), pH 7.4, Gibco 42360-032). For ATP-induced NLRP3 inflammasome activation, slices were incubated at 37°C on culture inserts (30-mm diameter, 0.4-μm pore size, Merck Millipore PICM0RG50) in six-well plates containing 1 ml of serum-free Dulbecco’s Modified Eagle’s Medium (DMEM; pH 7.4, Gibco 41965-039) with or without the addition of drug(s) for the first 3 hours, followed by application of 5 mM ATP for another 3 hours serving as a combined priming and activation stimulus (*47*). Supernatants were then collected and stored at −80°C until the IL-1β levels were measured via high-sensitivity enzyme-linked immunosorbent assay (ELISA), using Meso Scale Discovery Proinflammatory Panel 1 kits (Human K15049D and Mouse K15048D).

### Co-IP and Western blotting

Interactions between CLIC1 and moesin were studied by Co-IP in HEK293 cells recombinantly expressing both proteins, as well as in MACS-isolated microglia under endogenous conditions.

Plasmids: Adeno-associated virus (AAV) expression vectors with a CAG promoter and an internal ribosomal entry site (IRES)–eGFP cassette were used for bicistronic expression in HEK cells. CLIC1 [National Center for Biotechnology Information (NCBI) accession no. BC004658] and moesin (NCBI accession no. S47577.1) cDNAs were amplified from a mouse brain cDNA library, cloned into TOPO-TA vectors (Thermo Fisher Scientific), verified by sequencing, and subcloned into AAV backbones via Gibson Assembly (NEB). The CLIC1 reverse primer additionally included a FLAG tag sequence, yielding the constructs pAAV.CAG-CLIC1-flag-IRES-eGFP-wpre3 and pAAV.CAG-moesin-IRES-eGFP-wpre3. To mimic the active, open conformation of phosphorylated moesin, phosphomimetic mutants (T558D, T235D, and T558D) were generated by site-directed mutagenesis using overlapping polymerase chain reaction fragments and Gibson Assembly, resulting in pAAV.CAG-moesin-T558D-IRES-eGFP-wpre3 and pAAV.CAG-moesin-T235D, T558D-IRES-eGFP-wpre3. Constructs were sequence-verified and used for transfection in HEK cells to assess protein interactions via Co-IP.

HEK293T cells (American Type Culture Collection, CRL-11268) were cultured in DMEM supplemented with 10% fetal calf serum and 1% penicillin/streptomycin at 37°C and 5% CO_2_. For the transfection of 350,000 cells, 2 μg of each plasmid (total of 4 μg) and 12 μl of polyethylenimine (1 mg/ml) were incubated for 20 min and added to the cells. The medium was changed to fresh medium after 4 hours, and the cells were lysed 24 hours later.

Cell lysate preparation: Cells were washed once in ice-cold PBS and lysed in cold Co-IP buffer [50 mM tris, pH 7.8, 150 mM NaCl, 1% NP-40, 2 mM dithiothreitol (DTT), and 5% glycerol] or radioimmunoprecipitation assay buffer [50 mM tris-HCl, pH 7.4, 150 mM NaCl, 0.5% sodium deoxycholate, 1% NP-40, 0.1% SDS supplemented with protease inhibitors (Calbiochem Set III), and phosphatase inhibitors (1 mM Na_2_MO_4_, 1 mM NaF, 20 mM β-glycerophosphate, 1 mM Na_3_VO_4_, and 500 nM cantharidin)]. Homogenates were centrifuged at 10,000*g* for 15 min, and the supernatant was collected for further analysis. A sample of the total cell lysate was transferred to a tube containing ROTI Load 1 sample buffer (ROTH). The leftover lysate was incubated with 10 μl of anti-FLAG M2 affinity gel (Sigma-Aldrich) for 2 hours. Immunoprecipitates were washed three times in Co-IP buffer and eluted in ROTI Load 1 sample buffer (ROTH). For immunoprecipitation of CLIC1 in MACS-isolated microglia, cells were lysed in PBS containing 1% NP-40, 2 mM DTT, and 2 mM EDTA supplemented with protease and phosphatase inhibitors. Homogenates were centrifuged at 10,000*g* for 15 min, and the supernatant was collected for further immunoprecipitation. Lysates were incubated overnight with 40 μg of CLIC1 antibody (tmCLIC1omab) at 4°C followed by a 2-hour incubation with 100 μl of Pierce Protein A/G Agarose (Thermo Fisher Scientific).

SDS–polyacrylamide gel electrophoresis (SDS-PAGE) and Western blotting: Immunoprecipitates were washed three times in lysis buffer and eluted in ROTI Load 1 sample buffer (ROTH). On average, 15 to 30 μg of protein was loaded on an SDS-PAGE gel. Western blot analysis was performed as previously described (*99*) using mouse anti-CLIC1 (SC-271051, 1:1000, Santa Cruz) or rabbit anti-CLIC1 (D7D6H, #53424, 1:1000, Cell Signaling), rabbit anti-moesin (ab52490, 1:1000, Abcam), rabbit anti-ERM (3142, 1:1000, Cell Signaling), mouse anti-GAPDH 6C5 (CB1001, 1:1000, Millipore), and mouse anti-β-actin (AC-15, #GTX26276, 1:1000, Genetex). Quantification of band densities was performed using Fiji. The area of the band and the mean gray value were measured to obtain a relative density. For relative quantifications, measurements were normalized to glyceraldehyde-3-phosphate dehydrogenase (GAPDH) loading control.

### Statistics

Statistical analyses were performed using OriginPro 2023 software. For normally distributed data, either two-tailed one-sample or two-sample Student’s *t* test was applied, depending on the experimental design. For nonnormally distributed data, the Mann-Whitney *U* test (for independent data) or Wilcoxon signed-rank test (for dependent data) was used. Normality of the data was determined using Anderson-Darling and Shapiro-Wilk tests. The *F* test was used to test for equality of variances, and Welch’s *t* tests were applied when variances were unequal. Group comparisons were made using analysis of variance (ANOVA) or Kruskal-Wallis test, followed by appropriate post hoc tests as indicated for each analysis in the corresponding figure legend. An estimate of an appropriate sample size for a standard experiment was as follows: for a control response of 100%, a typical response standard deviation of 25%, an effect size of 50%, a power of 80%, and a significance level of *P* < 0.05, at least six cells are required in both groups (www.biomath.info/power/ttest.htm). Actual numbers may vary for different experiments depending on the effect size and standard deviation. Significance was set to an α level of 0.05. Data are presented as means ± SEM (standard error of the mean). Where appropriate, analyses were performed with the researcher blinded to the experimental conditions.
