# Supplementary material for: The chloride intracellular channel 1 (CLIC1) is essential for microglial morphodynamics and neuroinflammation
Source: Sci Adv. 2025 Oct 22;11(43):eads9181. doi: 10.1126/sciadv.ads9181 (PMC12542960; doi:10.1126/sciadv.ads9181)
Supplement: Supplementary file 1 — Figs. S1 to S6 Legends for tables S1 and S2 Legends for movies S1 to S6 [file sciadv.ads9181_sm.pdf]

Science Advances
AAAS

Supplementary Materials for

The chloride intracellular channel 1 (CLIC1) is essential for microglial morphodynamics and neuroinflammation

Ali Rifat *et al.*

Corresponding author: Christian Madry, christian.madry@charite.de

Sci. Adv. **11**, eads9181 (2025)
DOI: 10.1126/sciadv.ads9181

The PDF file includes:

Figs. S1 to S6
Legends for tables S1 and S2
Legends for movies S1 to S6

Other Supplementary Material for this manuscript includes the following:

Tables S1 and S2
Movies S1 to S6

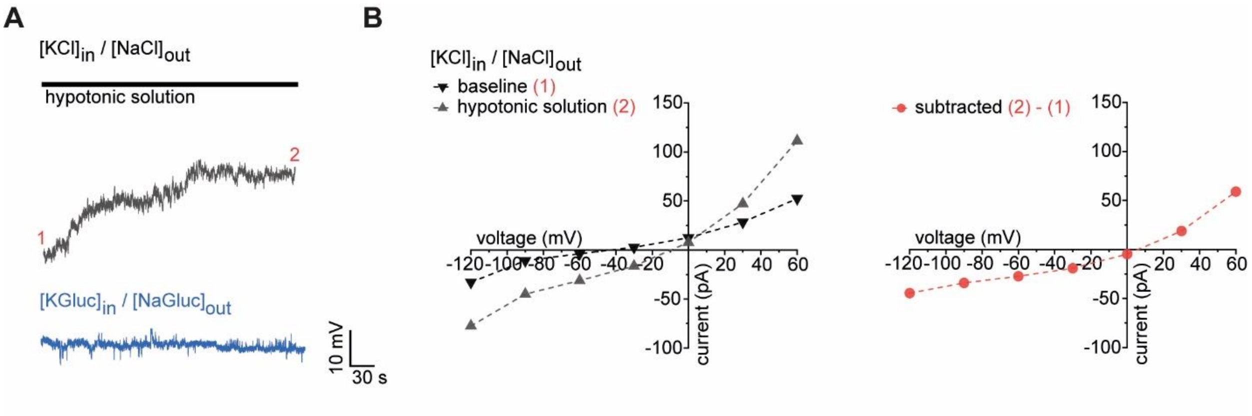

Fig. S1. Microglia generate outwardly rectifying membrane currents to hypotonic stimulation only in the presence of Cl^-. (A) Voltage response of microglia to hypotonic stimulation for Cl^--containing control (top) and Cl^--free conditions (bottom). Note the lack of change in membrane voltage in the absence of Cl^- (blue trace). (B) Current-to-voltage relationships from -120 mV to +60 mV of a single microglia before (baseline) and after exposure to hypotonic solution (left), and respective voltage dependence of the net current induced by the hypotonic stimulus by subtracting (2) − (1) (right). Note the Cl^- reversal potential of ~ 0 mV due to the equal Cl^- concentration in the intra- and extracellular solution.

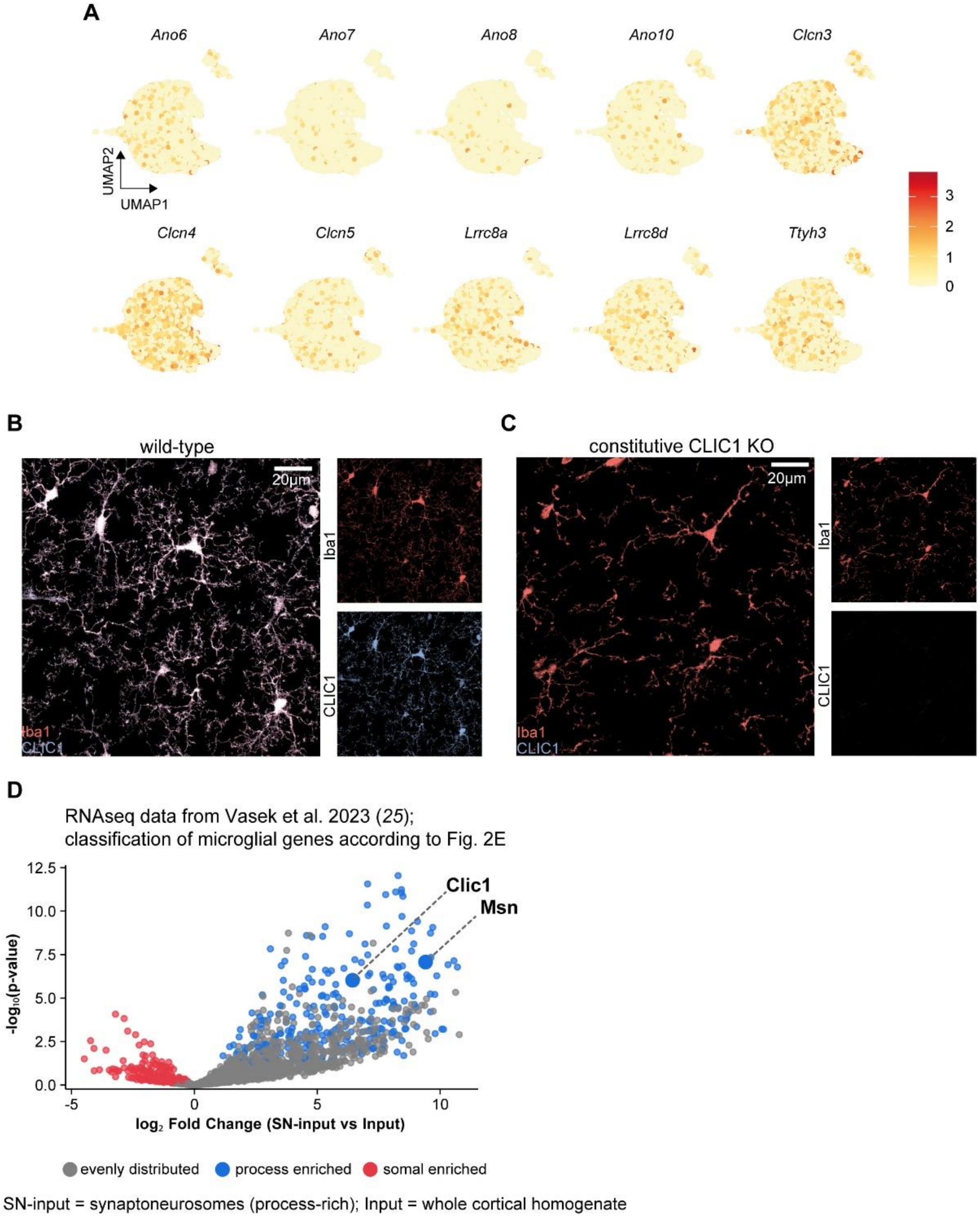

Fig. S2. *Clic1* is the dominantly expressed chloride channel in microglia and is enriched in processes. (A) UMAPs of selected Cl^- channel gene expression across individual murine microglia, complementing expression profiles in Fig. 2C, D. **(B, C)** Specimen confocal images of Iba1-positive microglia (red) in wild-type **(B)** and constitutive CLIC1 KO **(C)** mice co-localizing with CLIC1 (blue) immunoreactivity. Merged images and corresponding single-channel views are shown. Absence of CLIC1 signal in CLIC1-deficient mice confirms antibody specificity and gene knockout. **(D)** Volcano plot showing the distribution of microglia-specific transcripts across synaptoneurosome (SN) and total cortical input fractions, from Vasek et al. (*25*). The SN fraction contains microglial processes. Transcripts were classified as somal (red), evenly distributed (grey), or process-enriched (blue), based on TRAP-RNA-seq analysis from *Cx3cr1*$^{\text{CreERT2}}$; *Rpl10a*^{EGFP}

mice and subsequent localization scoring (see Vasek et al. (*25*), Fig. 2E). Note that *Clic1* and *Msn* are among the most prominently process-enriched genes.

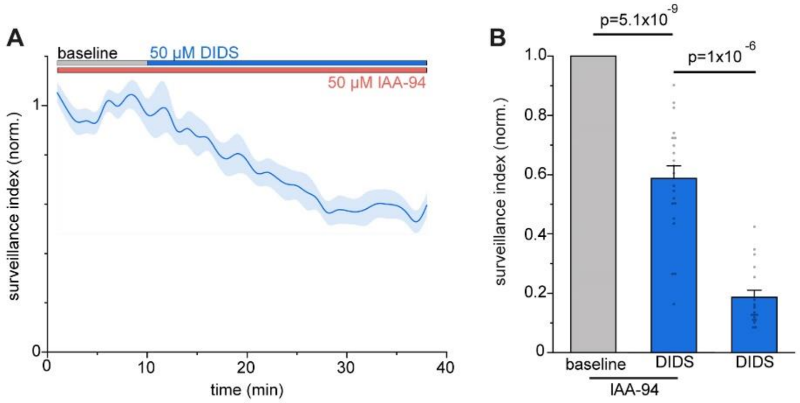

Fig. S3. DIDS-induced decrease in surveillance is much weaker in the presence of IAA-94. **(A)** Time course of surveillance of microglia pre-treated with IAA-94 before (baseline) and with application of DIDS. **(B)** Comparison of surveillance indices for DIDS in the presence of IAA or when applied alone, normalized to the first 10 min of baseline. Data information: Data indicate mean ± SEM. Dots on bars show number of cells. *P*-values are from paired (DIDS in presence of IAA) and unpaired (DIDS alone) Student's t-test.

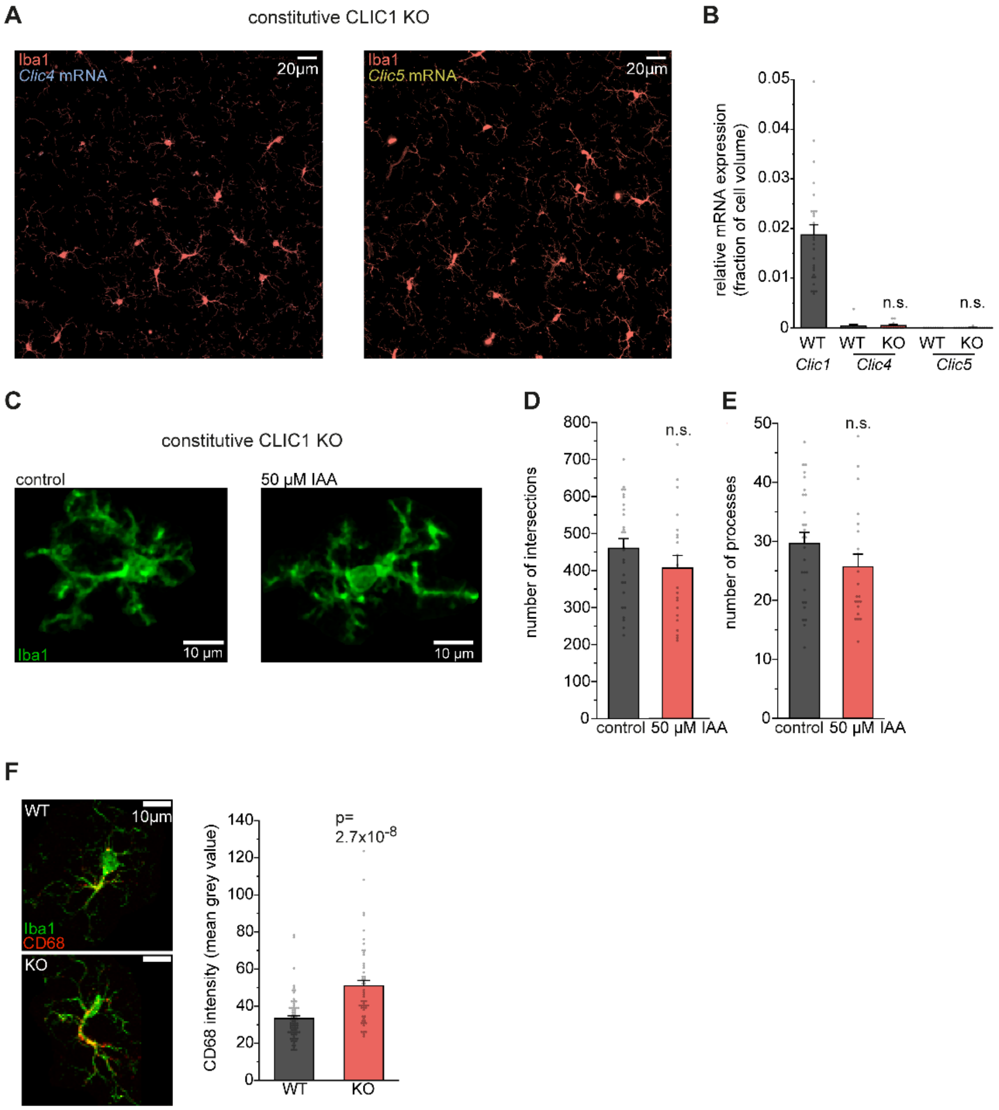

Fig. S4. Lack of compensatory *Clic* upregulation and abolishment of IAA-94 effects on microglial morphology in CLIC1 KO mice. (A) Specimen images visualizing *Clic4* (left) and *Clic5* (right) mRNA by RNAscope *in situ* hybridization co-labeled for microglia by Iba1 immunofluorescence in brain sections from constitutive CLIC1 KO mice. *Clic4* and *Clic5* mRNA signals are shown within areas of Iba1-positive immunoreactivity. **(B)** Quantification of mRNA expression in microglia from wild-type and CLIC1 KO mice, shown as mRNA per total cell volume. **(C)** Specimen images showing Iba1-labeled microglia in CLIC1 KO mice without (control) and with application of 50 µM IAA-94. **(D, E)** Comparison of microglial ramification for Sholl analysis derived total number of intersections **(D)** and processes **(E)** of microglia from CLIC1 KO mice in the absence (control) and presence of IAA. **(F)** Left: Specimen images showing Iba1 and CD68 labeled microglia in WT and CLIC1 KO mice. Right: Quantification of the intensity of CD68 immunoreactivity per cell for WT and CLIC1 KO. Data information: Data indicate mean ± SEM. Dots on bars show number of cells. *P*-values are from unpaired Student's t-test (D) and Mann-Whitney *U* test (B, E, F).

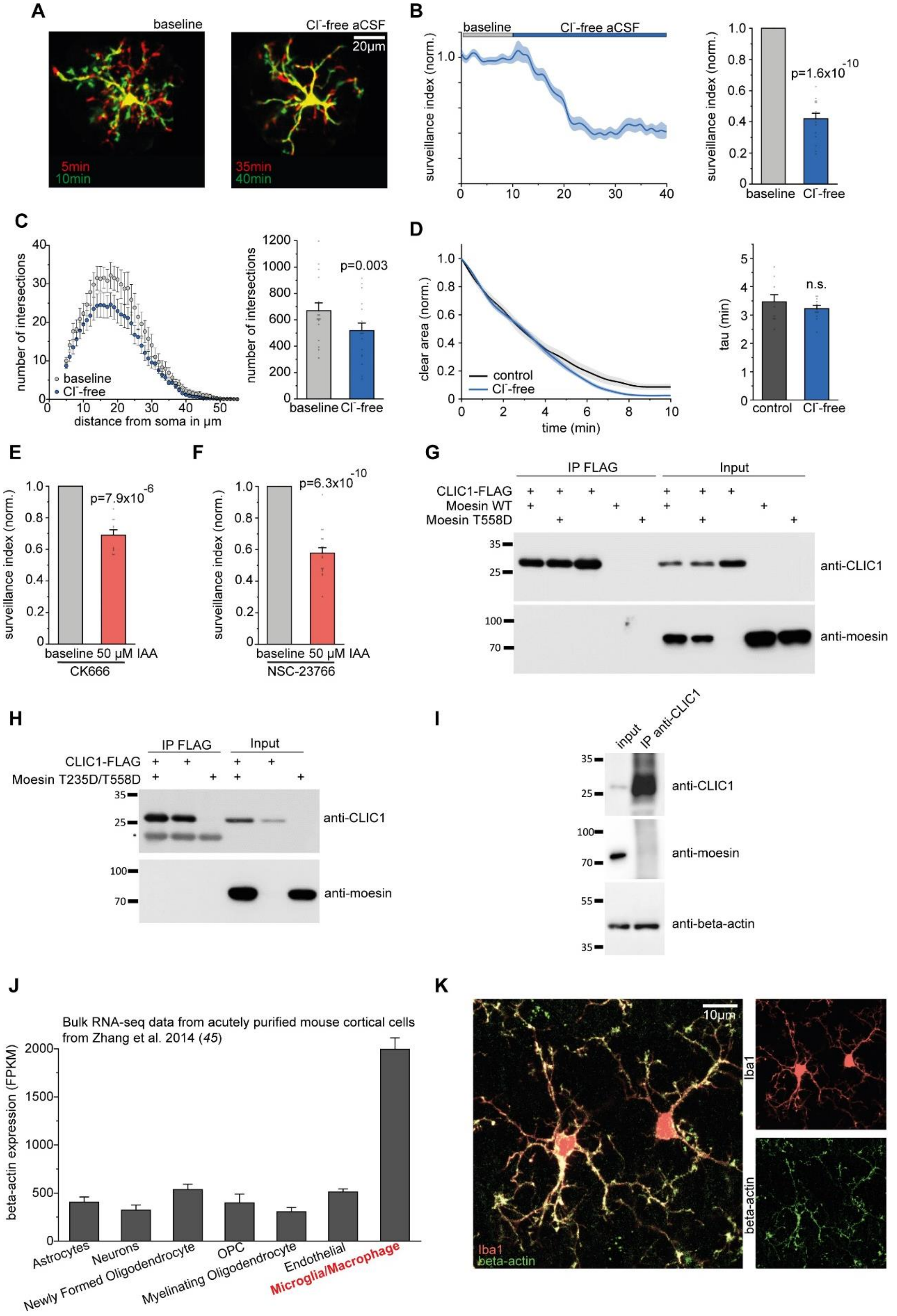

A
baseline
Cl⁻-free aCSF
20µm
5min
10min
35min
40min
B
baseline
Cl⁻-free aCSF
surveillance index (norm.)
p=1.6x10-10
baseline
Cl⁻-free
C
number of intersections
distance from soma in µm
baseline
Cl⁻-free
p=0.003
D
clear area (norm.)
time (min)
control
Cl⁻-free
tau (min)
n.s.
E
surveillance index (norm.)
p=7.9x10-6
baseline
50 µM IAA
CK666
F
p=6.3x10-10
NSC-23766
G
IP FLAG
Input
CLIC1-FLAG
Moesin WT
Moesin T558D
anti-CLIC1
anti-moesin
H
Moesin T235D/T558D
I
input
IP anti-CLIC1
anti-beta-actin
J
Bulk RNA-seq data from acutely purified mouse cortical cells
from Zhang et al. 2014 (45)
beta-actin expression (FPKM)
Astrocytes
Neurons
Newly Formed Oligodendrocyte
OPC
Myelinating Oligodendrocyte
Endothelial
Microglia/Macrophage
K
10µm
Iba1
beta-actin

Fig. S5. Effects of Cl^- depletion and involvement of actin cytoskeletal modulation in CLIC1-dependent microglial morphodynamics. (A) Superimposed images of microglia acquired 5 min apart visualizing surveillance before and under Cl^--free conditions (colors as in Fig. 1A). **(B)** Left: Time course of microglial surveillance before and during exposure of Cl^--free conditions, normalized to baseline (with Cl^-). Right: Comparison of mean effect of Cl^- depletion on surveillance. **(C)** Comparison of microglial ramification by Sholl analysis (left) and quantification of the total number of intersections (right) during baseline and after Cl^- depletion. **(D)** Left: Analysis of microglial chemotaxis plotted as the progressing decrease in clear area not covered by microglial processes over time. Clear area is normalized to that at chemotaxis onset. Right: Comparison of time constants of the decay. **(E, F)** Analysis of the mean effects of 50 µM IAA-94 in the presence of the Arp2/3 blocker CK-666 (200 µM) **(E)** or the Rac GTPase blocker NSC-23766 (100 µM) **(F)** on microglial surveillance. **(G)** Co-immunoprecipitation assay upon expression of CLIC1-FLAG and moesin wild-type (WT) or moesin T558D in HEK293T cells. Following anti-FLAG immunoprecipitation (left), co-precipitates were analyzed with anti-moesin antibodies. The expression of each plasmid was verified in the total cell lysate (input, right). **(H)** As in (G) CLIC1-FLAG and double mutant moesin T235D/T558D were co-expressed in HEK293T cells and co-immunoprecipiation was verified using CLIC1 and moesin antibodies. Star (*) depicts light chain of IgG antibody molecule. **(I)** Co-immunoprecipitation of endogenous CLIC1 from lysates of acutely isolated mouse microglia using mouse anti-CLIC1 antibody (tmCLIC1omab®). Co-precipitates were analyzed by immunoblot using antibodies against moesin, beta-actin, and CLIC1. Molecular weight protein ladder for G, H, I is in kilodaltons. (J) Bulk RNA-seq data from acutely purified mouse cortical cells from Zhang et al. (*45*) showing beta-actin (*Actb*) expression across different CNS cell types. Note highest expression in microglia/macrophages. (K) Representative confocal images of mouse microglia co-labeled for Iba1 (red) and beta-actin (green). Merged image and single channels are shown. Beta-actin signal is enriched in microglial processes. Data information: Data indicate mean ± SEM. Dots on bars show number of cells, except for (I), where each dot represents a single chemotactic event. Data are from 3 (D-F) and 12 mice (I). *P*-values are from paired (B, C) and unpaired (E, F) Student's t-tests and Welch's t-test (D).

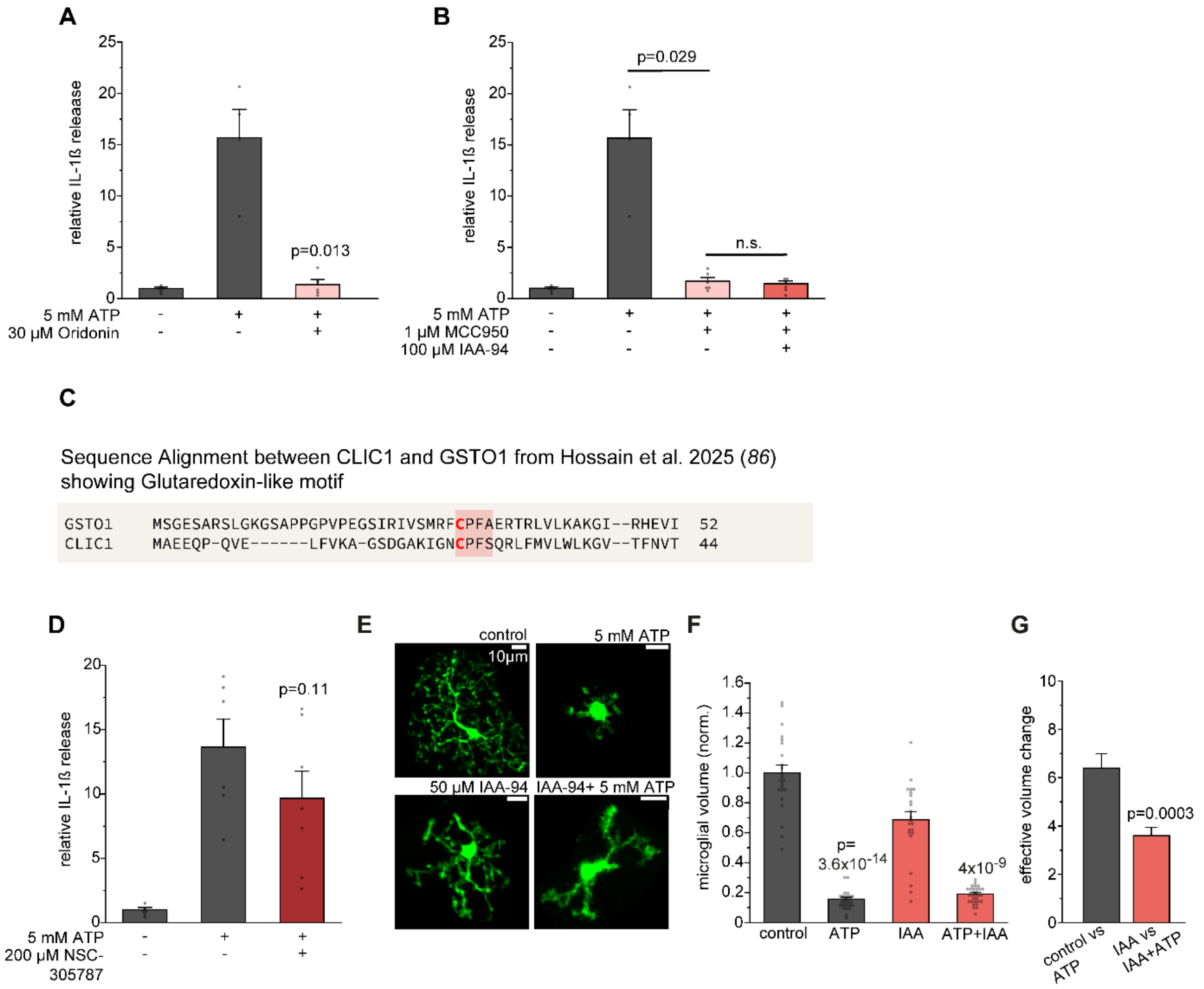

Fig. S6. Implications of CLIC1 on NLRP3 activation. **(A)** Oridonin, an alternative covalent-binding NLRP3 inhibitor, almost completely suppressed ATP-induced IL-1β release. **(B)** Co-application of MCC950 and IAA-94 did not produce an additive effect on IL-1ß release compared to MCC950 alone. **(C)** Sequence alignment of GSTO1 and CLIC1 adapted from Hossain et al. (*86*), highlighting the common glutaredoxin-like motif. **(E)** Specimen images of Iba1-labeled microglia before (control) and after purinergic stimulation (5 mM ATP) in the absence or presence of 50 µM IAA-94. **(F)** Comparison of mean changes in cell volume of non-activated and ATP-activated microglia with and without CLIC1 blockade by IAA-94. **(G)** Effective fold changes in microglial cell volume comparing control to ATP treated cells, and IAA to IAA with ATP treated cells. Data information: Data indicate mean ± SEM. Dots on bars show number of slices (A, B, D) or number of cells (F). Data are from 4 (A, B, D) and 3 (F–G) mice, respectively. *P*-values are from Welch's t-test (A, F, G), Welch's ANOVA with Games-Howell post hoc test (B) and unpaired Student's t-tests (D).

Suppl. Table 1 / Excel 1: Cluster-specific marker gene expression in human microglia.

Suppl. Table 2 / Excel 2: Source data for figures and statistical analysis.

Supplementary Movie S1: Two-photon time-lapse imaging of microglial surveillance (control).

Supplementary Movie S2: Two-photon time-lapse imaging of microglial surveillance (DIDS).

Supplementary Movie S3: Two-photon time-lapse imaging of microglial surveillance (IAA-94)

Supplementary Movie S4: Two-photon time-lapse imaging of microglial surveillance (A9C).

Supplementary Movie S5: Two-photon time-lapse imaging of microglial chemotaxis (control).

Supplementary Movie S6: Two-photon time-lapse imaging of microglial chemotaxis (IAA-94).
